# Human GTPBP5 is involved in the late stage of mitoribosome large subunit assembly

**DOI:** 10.1093/nar/gkaa1131

**Published:** 2020-12-07

**Authors:** Miriam Cipullo, Sarah F Pearce, Isabel G Lopez Sanchez, Shreekara Gopalakrishna, Annika Krüger, Florian A Rosenberger, Jakob D Busch, Xinping Li, Anna Wredenberg, Ilian Atanassov, Joanna Rorbach

**Affiliations:** Department of Medical Biochemistry and Biophysics, Division of Molecular Metabolism, Karolinska Institutet, Solnavägen 9, 171 65 Solna, Sweden; Max Planck Institute Biology of Ageing - Karolinska Institutet Laboratory, Karolinska Institutet, Stockholm, Sweden; Department of Medical Biochemistry and Biophysics, Division of Molecular Metabolism, Karolinska Institutet, Solnavägen 9, 171 65 Solna, Sweden; Max Planck Institute Biology of Ageing - Karolinska Institutet Laboratory, Karolinska Institutet, Stockholm, Sweden; Department of Medical Biochemistry and Biophysics, Division of Molecular Metabolism, Karolinska Institutet, Solnavägen 9, 171 65 Solna, Sweden; Centre for Eye Research Australia, Royal Victorian Eye and Ear Hospital, 32 Gisborne Street, East Melbourne, 3002 Victoria, Australia; Department of Medical Biochemistry and Biophysics, Division of Molecular Metabolism, Karolinska Institutet, Solnavägen 9, 171 65 Solna, Sweden; Max Planck Institute Biology of Ageing - Karolinska Institutet Laboratory, Karolinska Institutet, Stockholm, Sweden; Department of Medical Biochemistry and Biophysics, Division of Molecular Metabolism, Karolinska Institutet, Solnavägen 9, 171 65 Solna, Sweden; Max Planck Institute Biology of Ageing - Karolinska Institutet Laboratory, Karolinska Institutet, Stockholm, Sweden; Max Planck Institute Biology of Ageing - Karolinska Institutet Laboratory, Karolinska Institutet, Stockholm, Sweden; Department of Molecular Medicine and Surgery, Karolinska Institutet, Karolinska University Hospital, Solna (L1:00), 171 76 Stockholm, Sweden; Department of Mitochondrial Biology, Max-Planck-Institute for Biology of Ageing, Joseph-Stelzmann-Str. 9b, 50931 Cologne, Germany; Proteomics Core Facility, Max-Planck-Institute for Biology of Ageing, Joseph-Stelzmann-Str. 9b, 50931 Cologne, Germany; Department of Medical Biochemistry and Biophysics, Division of Molecular Metabolism, Karolinska Institutet, Solnavägen 9, 171 65 Solna, Sweden; Max Planck Institute Biology of Ageing - Karolinska Institutet Laboratory, Karolinska Institutet, Stockholm, Sweden; Proteomics Core Facility, Max-Planck-Institute for Biology of Ageing, Joseph-Stelzmann-Str. 9b, 50931 Cologne, Germany; Department of Medical Biochemistry and Biophysics, Division of Molecular Metabolism, Karolinska Institutet, Solnavägen 9, 171 65 Solna, Sweden; Max Planck Institute Biology of Ageing - Karolinska Institutet Laboratory, Karolinska Institutet, Stockholm, Sweden

## Abstract

Human mitoribosomes are macromolecular complexes essential for translation of 11 mitochondrial mRNAs. The large and the small mitoribosomal subunits undergo a multistep maturation process that requires the involvement of several factors. Among these factors, GTP-binding proteins (GTPBPs) play an important role as GTP hydrolysis can provide energy throughout the assembly stages. In bacteria, many GTPBPs are needed for the maturation of ribosome subunits and, of particular interest for this study, ObgE has been shown to assist in the 50S subunit assembly. Here, we characterize the role of a related human Obg-family member, GTPBP5. We show that GTPBP5 interacts specifically with the large mitoribosomal subunit (mt-LSU) proteins and several late-stage mitoribosome assembly factors, including MTERF4:NSUN4 complex, MRM2 methyltransferase, MALSU1 and MTG1. Interestingly, we find that interaction of GTPBP5 with the mt-LSU is compromised in the presence of a non-hydrolysable analogue of GTP, implying a different mechanism of action of this protein in contrast to that of other Obg-family GTPBPs. GTPBP5 ablation leads to severe impairment in the oxidative phosphorylation system, concurrent with a decrease in mitochondrial translation and reduced monosome formation. Overall, our data indicate an important role of GTPBP5 in mitochondrial function and suggest its involvement in the late-stage of mt-LSU maturation.

## INTRODUCTION

Ribosomes are conserved macromolecular structures that catalyse protein synthesis in all organisms. Mitochondria retain their own translation machinery, consisting of the mitoribosomes, which produces 13 essential polypeptide components of the oxidative phosphorylation (OxPhos) complexes. Human mitoribosomes sediment as 55S particles and comprise the 39S large subunit (mt-LSU), formed by 16S mt-rRNA, mt-tRNA^Val^ and 52 mitoribosomal proteins (MRPs), and the 28S small subunit (mt-SSU), formed by 12S mt-rRNA and 30 MRPs (1–3). Mitoribosomes exhibit unique features due to the acquisition of both mitochondrial-specific proteins and mitochondrial N- and C-terminal extensions of the universal ribosomal proteins, and drastic reduction in mt-rRNAs size. Together, these differences result in a higher protein:rRNA ratio when compared to bacterial ribosomes, reflecting a functional and structural divergence that remains to be fully understood. Furthermore, the dual genetic origin of the mitoribosome (rRNAs and tRNA are encoded by the mitochondrial DNA (mtDNA) whereas all 82 MRPs are encoded in the nucleus) suggests complex mechanisms are needed for coordinated production and assembly of mitoribosomal components (4).

Mutations in both MRP genes and factors which contribute to mitoribosome biogenesis have been reported to cause severe mitochondrial disorders and therefore, understanding the molecular mechanisms underlying these processes is of crucial importance (5,6). Recent advances in cryo-electron microscopy (cryo-EM) have allowed for a characterization of the complexity of the mitoribosome structure (2), including association with a few accessory factors involved in mitoribosome maturation, such as MALSU1, L0R8F8 and mt-ACP (mitochondrial acyl-carrier protein) (7). However, a complete understanding of which factors are involved, and how such accessory proteins co-ordinate assembly, remains to be determined.

GTP-binding proteins (GTPBPs) are important regulatory proteins present in all domains of life, with functions implicated in a variety of cellular processes, including receptor signaling, intracellular signal transduction, cytoskeleton organization and protein synthesis. These proteins are a family of NTPases defined as molecular switches, named as such as they are generally active when bound with GTP and inactive when bound with GDP. GTP binding and hydrolysis take place via the G-domain, consisting of five conserved motifs (G1–G5), which is present in all GTPases and conserved across species (8–10).

Over the past decades, GTPBPs have been extensively studied in bacteria and represent one of the major groups of ribosomal assembly factors (11,12). Additionally, the repertoire of GTPBPs known to function in humans in mitoribosome assembly has been increasing in recent years. NOA1 (C4orf14) was shown to localize to mitochondria, where it was found to bind to the mt-SSU in a GTP-dependent manner, with the depletion of NOA1 leading to a mitochondrial translation defect (13,14). Era-like 1 (ERAL1) also associates with the mt-SSU, binding directly to 12S mt-rRNA (15,16). Another GTPase, MTG1 (GTPBP7), couples mt-LSU maturation with the formation of an intersubunit bridge, acting as a quality-control checkpoint in mitoribosome assembly (17,18). In addition, GTPBP10 has been shown to act in the mt-LSU assembly process (19–21).

Bacterial GTPBPs belonging to the Obg-family have been reported to have a role in assembly of the 50S subunit in many prokaryotic species. The Obg-family belongs to the TRAFAC (translation-factors-related) class and can be divided in subfamilies of high molecular mass GTPBPs (Obg, EngD, DRG, NOG). Obg proteins possess a highly conserved glycine-rich N-terminal domain in addition to the highly conserved GTP-binding domain and a C-terminal domain (22,9). In *Escherichia coli*, the recently resolved structure of the 50S–ObgE–GMPPNP complex has revealed that ObgE is an important factor in the final stages of the LSU assembly, acting as a quality-control checkpoint (23). Furthermore, in *S. cerevisiae*, Obg-family protein Mtg2p localizes to mitochondria, and has been reported to participate in the assembly of the 54S subunit (24).

In humans, two Obg-family proteins are present, GTPBP10 and GTPBP5 (Mtg2, ObgH1), both of which have previously been shown to localize to mitochondria. Ablation of GTPBP10 has been shown to lead to a severe mitochondrial translation defect and the factor is likely to be involved in the late stages of mt-LSU maturation (19–21). In a similar manner to the interaction of ObgE with the bacterial large subunit (23), interaction of GTPBP10 with the mt-LSU was found to be stabilized by the incubation with non-hydrolysable analogue GMPPNP (19).

In contrast to GTPBP10, characterization of GTPBP5 function has been limited. In addition to revealing mitochondrial localization of GTPBP5, work by Kotani *et al.* (17) showed that GTPBP5 has the capacity to co-sediment with the mt-LSU *in vitro*, through sucrose gradient centrifugation analysis. However, knock-down of *GTPBP5* using siRNA had no detectable detrimental effect on the OxPhos system or mitochondrial translation.

Our study herein, builds upon the initial characterization performed by Kotani *et al.* to show that GTPBP5 interacts with the mt-LSU in cultured HEK293T cells. Our interactome analysis reports a specific interaction of GTPBP5 with several late-stage mt-LSU assembly factors. Surprisingly, we find that GTP hydrolysis is required for binding of GTPBP5 to the mt-LSU, evidenced by the loss of GTPBP5-mt-LSU interaction in the presence of a non-hydrolysable analog of GTP, suggesting a different mechanism of action from GTPBP10. The loss of GTPBP5 causes a severe defect in OxPhos function, associated with a reduction in the synthesis rate of mtDNA-encoded proteins and decreased monosome formation.

While we prepared the revision of this work, similar findings were reported by Maiti *et al.* (25). Our study, together with Maiti *et al.*, extend on the previously limited knowledge of GTPBP5 function, to implicate it in the assembly of the human mitoribosome.

## MATERIALS AND METHODS

### Generation and maintenance of Flp-In T-Rex stable mammalian cell lines

The Flp-In T-Rex human embryonic kidney 293 (HEK293T) cell line (Invitrogen) was used to generate stable mammalian cell lines that allow for doxycycline-inducible expression of C-terminal FLAG-tagged GTPBP5 (GTPBP5::FLAG) in a dose-dependent manner. GTPBP5 cDNA was obtained from the hORFeome Database (Internal ID:12579) and subsequently cloned into pcDNA5/FRT/TO. Primers used for cloning of GTPBP5 and GTPBP5 mutant are listed in Supplementary Table S1. HEK293T cells were cultured in DMEM (Dulbecco's modified Eagle's medium) supplemented with 10% (v/v) tetracycline-free fetal bovine serum (FBS), 2 mM Glutamax (Gibco), 1× Penicillin/Streptomycin (Gibco), 50 μg/ml uridine, 10 μg/ml Zeocin (Invitrogen) and 100 μg/ml blasticidin (Gibco) at 37°C under 5% CO_2_ atmosphere. Prior to transfection, cells were seeded in a six-well plate and grown in culture medium lacking selective antibiotics. Cells were then transfected with pcDNA5/FRT/TO-GTPBP5::FLAG, and pOG44 constructs using Lipofectamine 3000 according to manufacturer's instructions. At 48 h post-transfection cells were selected by addition of hygromycin (100 μg/ml, Invitrogen) and blasticidin (100 μg/ml) to the culture medium. After two to three weeks, selected colonies were picked and cultured as single clonal populations. To confirm inducible expression of GTPBP5, cells were incubated with 100 ng/ml doxycycline, and cell pellets were harvested for western blot analysis 24 or 48 h later.

For cell growth measurements, cells were seeded at a density of 2 × 10^4^ in six-well plates and grown in the presence or absence of 50 ng/ml of doxycycline in glucose-free DMEM supplemented with 0.9 g/l galactose, 10% (v/v) FBS, 1 mM sodium pyruvate, 2 mM Glutamax and 1× Penicillin/Streptomycin. Cell number was measured at 48 hourly intervals with EVE™ Automated Cell Counter (NanoEnTek).

For SILAC-based quantitative proteomic analysis, cells were grown in Iscove's modified Dulbecco's medium (IMDM) supplemented with ‘heavy’ (^15^N- and ^13^C-labelled) or ‘light’ Arg, Lys and 10% dialysed FCS (Thermo Scientific HyClone).

### Generation of GTPBP5 knock-out cell line

GTPBP5 is encoded by the *MTG2* gene. Generation of the HEK293T knock-out cell line for *MTG2* was performed using CRISPR/Cas9 system as described in Ran *et al.* (26). Two pairs of gRNAs targeting exon 1 of *MTG2* were designed and cloned into the pSpCas9(BB)-2A-Puro (pX459) V2.0 vector to generate out-of-frame deletions. Cells were transfected with the pX459 variants using Lipofectamine 3000 according to manufacturer's recommendations. Transfected cell populations were selected by puromycin treatment at a final concentration of 1.5 μg/ml for 48 h. Subsequent to this, cells were diluted to achieve single-cell derived clones on 96-well plates. Resultant clones were screened by Sanger sequencing to assess knockout and loss of GTPBP5 in selected clones was confirmed by western blotting.

### Immunocytochemistry analysis

Human 143B osteosarcoma (HOS) cell line, HEK293T, HEK293T overexpressing GTPBP5::FLAG and GTPBP5^KO^ cells with re-expression of GTPBP5 (GTPBP5^RESCUE^) were used for immunocytochemistry (ICC) analysis. Cells were cultured in DMEM supplemented with 10% (v/v) FBS, 2 mM Glutamax and 1× Penicillin/Streptomycin at 37°C under 5% CO_2_ atmosphere. 143B cells were transiently transfected with pcDNA5/FRT/TO-GTPBP5::FLAG using Lipofectamine 3000 according to manufacturer's instructions, while HEK293T cells, HEK293T cells overexpressing GTPBP5::FLAG and GTPBP5^RESCUE^ cells were induced with 50 ng/ml of doxycycline 48 h prior the analysis.

The ICC experiment was performed on fixed cells as previously described (27). For 143B cells, TOM20 was used to detect the mitochondrial network while for the remaining cell lines MitoTracker Red CMXRos was supplemented to the medium for 15 min prior to fixation. Images were acquired using Zeiss LSM800 confocal microscope.

### Immunodetection of proteins

For immunoblot analysis, either 12 or 20 μg aliquots of total cell lysate protein were used to detect steady state levels of OxPhos complexes and mitoribosomal proteins, respectively. To detect GTPBP5, 20 μg of lysed isolated mitochondria was used. The lysates were subjected to SDS-PAGE and wet transferred onto PVDF membranes (Millipore) at 4°C for 1 h and 10 min at 300 mA. Blocking solution of 5% non-fat milk (Semper) in PBS-T (1× phosphate buffered saline supplemented with 1% Triton X-100) was applied to the membranes for 1 h at RT. The membranes were later incubated with specific primary antibodies in 5% milk in PBS-T overnight. Subsequently, membranes were washed three times with PBS-T and incubated with the HRP-conjugated secondary antibodies (GE Healthcare) in 5% milk in PBS-T for 1 h at RT and, after three washes with PBS-T, visualized using ECL (Bio-Rad). Supplementary Table S2 lists the antibodies used in this study.

### Immunoprecipitation of proteins

Isolation of mitochondria was performed from HEK293T cells expressing GTPBP5::FLAG as described in Rorbach *et al.* (28). Pelleted mitochondria were suspended in lysis buffer (Sigma-Aldrich, St. Louis, MO, USA) freshly supplemented with 1× PIC (cOmplete, Mini, EDTA-Free, Protease Inhibitor Cocktail, Roche) and 5 mM MgCl_2_. For specific analysis, 5′-guanylyl-imidodiphosphate trisodium salt hydrate (GMPPNP) at a final concentration of 20 mM and RNase A at a final concentration of 20 μg/ml were also added. Immunoprecipitation was performed using the FLAG-immunoprecipitation (IP) kit (Sigma-Aldrich) following manufacturer's instructions. Wash and elution buffers were freshly supplemented with 5 mM MgCl_2_. Elution was performed using 3X FLAG peptide according to manufacturer's instructions.

### [^35^S]- metabolic labelling of mitochondrial proteins

To label newly-synthesized mtDNA-encoded proteins, cells were seeded into a six-well dish at 80–90% confluency. Cell lines overexpressing GTPBP5 or GTPBP5^S238A^ were induced with 50 or 0.5 ng/ml of doxycycline prior to the experiment. Firstly, two washing steps of 5 min each in methionine/cysteine-free DMEM were performed. Subsequently, cells were incubated with fresh methionine/cysteine-free DMEM supplemented with Glutamax 100× (Gibco), Sodium Pyruvate 100× (Gibco), 10% dialysed FBS and 100 μg/ml emetine (Sigma-Aldrich) for 20 min at 37°C. Labeling was performed with addition of 166,7 μCi/ml of EasyTag EXPRESS [^35^S] protein labelling mix (methionine and cysteine) (Perkin Elmer) for 30 min at 37°C. Following labelling, cells were washed with 1 ml of PBS three times and the final pellets were collected by centrifugation and stored at –20°C. Cells were lysed in 1× PBS–PIC with addition of 50 units of benzonase (Life Technologies) with incubation on ice for 20 min, followed by addition of SDS to 1% f.c. and further incubation on ice for 30 min. The suspended pellets were stored at –20°C for 1 h to ease lysis. After cell lysis, 30 μg total protein, assayed by DC assay (Bio-Rad), were separated on Bolt 12% Bis–Tris Plus (Invitrogen) SDS-PAGE gels. Gels were then incubated in Imperial Protein Stain (Thermo Fisher) for 1 h at RT and with fixing solution (20% methanol, 7% acetic acid, 3% glycerol) for 1 h at RT. Finally, gels were vacuum-dried at 65°C for 2 h. The resultant gel was exposed to storage Phosphor screens and visualized with Typhoon FLA 7000 Phosphorimager.

### RNA isolation and northern blotting

Total RNA was isolated from GTPBP5^KO^ cell line, HEK293T cell line and sucrose gradient fractions using TRIzol (Invitrogen) according to manufacturer's instructions. RNA concentration was determined using NanoDrop ND-1000 UV-Vis Spectrophotometer (Thermo Scientific). For northern blot analysis, RNA was solubilized in NorthernMax™-Gly sample loading dye and 4 μg of RNA were resolved into 1.2 % (w/v) agarose gels containing 0.7% formaldehyde and 1× NorthernMax MOPS buffer (Life Technologies). Subsequently, gels were transferred onto Hybond-N+ membrane overnight. Membranes were then UV crosslinked and hybridized with DNA probes. [^32^P]-labelled DNA probes were prepared using the Prime-It II Random Primer Labelling Kit (Agilent Technologies) using 50 μCi of dCTP (PerkinElmer) according to manufacturer's instructions. Primers used to prepare DNA templates for [^32^P]-labelled DNA probes are listed in Supplementary Table S3. Following probing, membranes were washed with 1× SSC buffer (150 mM NaCl, 15 mM tri-sodium citrate (pH 7.0)). Probed membranes were exposed to a storage Phosphor screen and visualized using Typhoon FLA 7000 Phosphorimager.

### Sucrose gradient centrifugation analysis

Isolation of mitochondria was performed as described in Richter *et al.* (29). 1 mg of mitochondria were lysed in lysis buffer (10 mM Tris–HCl pH 7.5, 100 mM KCl or 50 mM KCl, 20 mM MgCl_2_, 1× PIC, 260 mM sucrose, 1% Triton X-100) freshly supplemented with 0.4 U/μl final concentration of RNase Block Ribonuclease Inhibitor (Agilent), then loaded onto a linear sucrose gradient (10–30% (w/v), 11 ml total volume) in 1× gradient buffer (20 mM Tris–HCl pH 7.5, 100 mM KCl or 50 mM KCl, 20 mM MgCl_2_, 1× PIC) and then centrifuged for 15 h at 79 000 × g at 4°C (Beckman Coulter SW41-Ti rotor). A total of 25 fractions with a volume of 450 μl each were collected via pipetting from the top of the gradient, and 15 μl of each fractions 3–17 inclusive were used for western blot analysis. For fractions 1 and 2, and for fractions 18 and 19, 7.5 μl of each fraction were combined and resolved together.

For SILAC-based proteomics, HEK293T or GTPBP5^KO^ cells and GTPBP5^RESCUE^ or GTPBP5^KO^ cells were grown in ‘heavy’ or ‘light’ labelled media for more than seven doublings as described in Van Haute *et al.* (30). Cell lines were pooled and mitochondrial isolation and sucrose gradient analysis were performed as described above. All buffers used for gradient analysis contained 50 mM KCl, instead of 100 mM KCl, to better preserve interaction with auxiliary factors.

### Analysis of mitochondrial respiratory complexes activity using BN-PAGE

BN-PAGE and in-gel activity measurements were performed on isolated mitochondria for Complex I, Complex IV and Complex V. Mitochondria were pelleted, resuspended in ACNA (1.5 M aminocaproic acid, 50 mM Bis–Tris pH 7.00) and quantified using Qubit. Following quantification, mitochondria were lysed using 4% (w/v) digitonin for 20 min on ice and subsequently centrifuged at maximum speed for 30 min at 4°C. 50 μg of proteins were then separated by Blue-Native Polyacrylamide Gel Electrophoresis (BN-PAGE) for about 5 h. The activity of mitochondrial respiratory complexes was measured in-gel by addition of buffers containing OxPhos substrates specific for Complex I (2 mM Tris–HCl, pH 7.4, 0.1 mg/ml NADH, 2.5 mg/ml iodonitrotetrazolium chloride), Complex IV (0.5 mg/ml DAB, 50 mM phosphate buffer pH 7.4, 1 mg/ml cytochrome *c*, 0.2 M sucrose, 20 μg/ml (1 nM) catalase) and Complex V (50 mM glycine, 5 mM MgCl_2_, 1% Triton X-100, 1.5 mM lead nitrate, 2 mM ATP).

### High-resolution mitochondrial respiration analysis

Mitochondrial respiration was measured by sequential addition of complex I and complex II substrates, uncoupler and inhibitors using an Oxygraph-2K oxygen electrode (Oroboros, Innsbruck, Austria) in MiR05 respiration buffer (110 mM d-sucrose, 20 mM taurine, lactobionic acid 60 mM, 10 mM KH_2_PO_4_, 3 mM MgCl_2_, 0.5 mM EGTA, 1 mg/ml fatty acid free bovine serum albumin (BSA), 20 mM HEPES pH 7.1).

Basal rate was first measured in intact cells (Basal) followed by permeabilization of cells using digitonin (10 μg/ml) and addition of complex I substrates glutamate (10 mM), malate (2 mM), pyruvate (2.5 mM) and ADP (2.5 mM) to measure complex I-linked respiration (CI-ADP; Complex I respiration—respiratory rate measured in the presence of complex I substrates (glutamate, malate, pyruvate) and ADP). Succinate (10 mM; complex II substrate) was then added to measure convergent complex I + II respiratory rate (CI+II-ADP; Complex I+II respiration—respiratory rate measured in the presence of complex I (glutamate, malate, pyruvate), complex II substrate (succinate) and ADP). Uncoupled, maximal respiration was subsequently measured in the presence of the uncoupler carbonyl cyanide m-chlorophenyl hydrazone (CCCP, 1.0 μM; Max U/C; Maximal respiratory rate—respiratory rate measured in the presence of complex I (glutamate, malate, pyruvate), complex II substrate (succinate), ADP and the uncoupler CCCP) and non-mitochondrial residual oxygen consumption rate (ROX) was measured after addition of rotenone (0.5 μM) and antimycin A (2.5 μM). Oxygen consumption rates were calculated using DatLab software and expressed as O_2_ flow (pmol/s/10^6^ cells). Differences between wild-type, KO and rescue cell lines were determined by two-tailed Student's *t* tests.

### Mass spectrometry analysis

#### Preparation of peptides from GTPBP5-IP for mass spectrometry

Proteins obtained from co-immunoprecipitations were prepared according to a modified protocol from Busch *et al.* (19) described below. Briefly, proteins obtained from GTPBP5::FLAG and HEK293T co-immunoprecipitations (*n* = 3) were resuspended in 200–300 μl of 0.6 M Guanidium chloride buffer (5 mM Tris–HCl pH 7.5, 0.1 mM Tris(2-carboxyethyl)phosphine (TCEP; ThermoFisher), 0.5 mM 2-chloroacetamid (Merck, cat. no. 8024120100)). Peptides were generated by overnight digest with 300 ng trypsin gold (Promega, cat. no. V5280, resuspended in 50 mM acetic acid) added directly to the resuspended protein samples. On the next day, peptides were desalted using home-made StageTips (Empore Octadecyl C18, 3M; (31)) and eluted with 80–100 μl of 60% acetonitrile/0.1% formic acid buffer. The eluted peptides were subsequently dried using a vacuum concentrator plus (Eppendorf) and resuspended with 0.1% formic acid for mass spectrometry.

#### Preparation of peptides from SILAC sucrose gradient experiment for mass spectrometry

Peptides from SILAC sucrose gradient centrifugation experiments were prepared from fractions 1 and 2 joined, 3 and 4 joined, and 5–17 individually. Collected fractions were precipitated in 20 × 100% ice-cold ethanol overnight at –20°C. Pelleted proteins were resuspended in 6 M GuHCl/Tris pH 8.0 solution and sonicated for 5 min at maximum output (10 s on/off cycles). After a 5 min incubation at RT, samples underwent a second round of sonication and were later centrifuged at maximum speed for 10 min. DTT at a final concentration of 5 mM was added to the obtained supernatants and incubated for 30 min at 55°C followed by incubation with 15 mM chloroacetamide for 15 min at RT in the dark. Prior to digestion, protein quantification was performed and trypsin (Pierce, trypsin protease MS-grade, Thermo Fisher Scientific) was added accordingly. Protein digestion was performed at 37°C overnight with mild shaking. After 12–14 h, trypsin was inactivated using 1.2% formic acid and samples were spun down at 3000 × g for 10 min at RT. Samples were desalted, using pre-packed C18 desalting columns (Thermo Fisher Scientific) previously equilibrated and washed respectively with 100% acetonitrile (ACN) and 0.5% formic acid, and eluted (0.5% formic acid, 50% ACN). Peptides were consequently dried using a SpeedVac Vacuum Concentrator and resuspended in 0.5% formic acid for mass spectrometry.

#### LC–MS/MS analysis

Peptides were separated on a 25 cm, 75 μm internal diameter PicoFrit analytical column (New Objective) packed with 1.9 μm ReproSil-Pur 120 C18-AQ media (Dr Maisch,) using an EASY-nLC 1200 (Thermo Fisher Scientific). The column was maintained at 50°C. Buffer A and B were 0.1% formic acid in water and 0.1% formic acid in 80% acetonitrile. Peptides were separated on a segmented gradient from 6% to 31% buffer B for 45 min and from 31% to 50% buffer B for 5 min at 200 nl/min. Eluting peptides were analysed on QExactive HF mass spectrometer (Thermo Fisher Scientific). Peptide precursor *m*/*z* measurements were carried out at 60 000 resolution in the 300–1800 *m*/*z* range. The ten most intense precursors with charge state from 2 to 7 only were selected for HCD fragmentation using 25% normalized collision energy. The *m*/*z* values of the peptide fragments were measured at a resolution of 30 000 using a minimum AGC target of 2e5 and 80 ms maximum injection time. Upon fragmentation, precursors were put on a dynamic exclusion list for 45 s.

For the analysis of the sucrose gradient fractions, peptides were separated using a segmented gradient from 6% to 31% buffer B and from 31% to 44% for 5 min. Eluting peptides were analysed on an Orbitrap Fusion Tribrid mass spectrometer (Thermo Fisher Scientific). Peptide precursor m/z measurements were carried out at 60 000 resolution in the 350–1500 *m*/*z* range. The 10 most intense precursors with charge state from 2 to 7 only were selected for HCD fragmentation using 27% normalized collision energy. The *m*/*z* values of the peptide fragments were measured at a resolution of 50 000 using a minimum AGC target of 2e5 and 86 ms maximum injection time. Upon fragmentation, precursors were put on a dynamic exclusion list for 45 s.

#### Protein identification and quantification

The raw data were analysed with MaxQuant version 1.5.3.8 (32) using the integrated Andromeda search engine (33). Peptide fragmentation spectra were searched against the canonical sequences of the human reference proteome (proteome ID UP000005640, downloaded September 2018 from UniProt). Methionine oxidation and protein N-terminal acetylation were set as variable modifications; cysteine carbamidomethylation was set as fixed modification. The digestion parameters were set to ‘specific’ and ‘Trypsin/P,’ The minimum number of peptides and razor peptides for protein identification was 1; the minimum number of unique peptides was 0. Protein identification was performed at a peptide spectrum matches and protein false discovery rate of 0.01. The ‘second peptide’ option was on. Successful identifications were transferred between the different raw files using the ‘Match between runs’ option. Label-free quantification (LFQ) (34) was performed using an LFQ minimum ratio count of two. LFQ intensities were filtered for at least two valid values in at least one group and imputed from a normal distribution with a width of 0.3 and down shift of 1.8. Protein quantification was performed using the t-test function in Perseus version 1.5.2.4 (35).

For SILAC labelling analyses, the raw data from the sucrose gradient fractions was analysed with MaxQuant version 1.6.1.0. Heavy labels were set to Arg10 and Lys8 and Label min. ratio count to 1. Match between runs was enabled between the same fraction number of the two sucrose gradients. For analyses, R version 3.5.2 and ggplot2 3.2.1 were used. Intensity values or normalized H/L ratios were extracted from the MaxQuant proteinGroups.txt file. Within each replicate, intensities of control (HEK293T or GTPBP5^RESCUE^) and GTPBP5^KO^ were normalized to each other against the median of the control, and then log2 transformed for plotting. Assembly factors associated with either the mt-SSU or the mt-LSU were manually curated.

### qPCR

For qPCR analysis, total RNA was extracted from HEK293T WT and GTPBP5^KO^ cells using TRIzol reagent according to manufacturer's instructions and RNA concentration was determined using Qubit RNA Broad Range assay kit (Thermo Fisher). Aliquots of 2.5 μg total RNA were treated with TURBO DNase (Thermo Fisher) to digest contaminating DNA, and 50% of treated RNA was transferred for cDNA synthesis using the High-Capacity cDNA Reverse Transcription kit (Thermo Fisher). Resultant cDNA was diluted 1:20 with nuclease free water and 1% of cDNA was used in each 10 μl qPCR reaction to assess steady-state levels of mt-mRNAs and mt-rRNAs. Quantitative PCR was performed using the QuantStudio 6 Flex Real-Time PCR System (Applied Biosystems) using the TaqMan Universal PCR Master Mix (Applied Biosystems) in combination with TaqMan probes labeled with FAM (6-carboxyfluorescein) reporter (Applied Biosystems) (Full list of probes used in this study are found in Supplementary Table S4). To determine steady-state levels of mt-mRNAs and mt-rRNAs, the 2^−ΔΔCt^ method was applied to determine fold change of mt-RNAs relative to the mRNA level endogenous house-keeping control ACTB. Data analysis was performed using Microsoft Excel software. Data represented combination of four biological replicates and error bars represents standard error of the mean (SEM).

### Statistics

The data represented from IVT, oxygraphy, growth curve and quantification analysis are derived from the mean values. Error bars represent the standard deviation. Statistical analysis was done by a two-tailed, unpaired-unequal variance Student's *t*-test. The significance threshold was set at *P* < 0.05; indicated as * for *P* < 0.05, ** for *P* < 0.01 and *** for *P* < 0.001.

## RESULTS

### GTPBP5 interacts with the mt-LSU and late-stage assembly factors

GTPBP5 has previously been described as a mitochondrial protein in human cells (17), a finding which we were able to reproduce via confocal microscopy, by confirming mitochondrial localization of an exogenously expressed GTPBP5 protein with a C-terminal FLAG tag (GTPBP5::FLAG) in 143B human osterosarcoma (HOS) cells (Supplementary Figure S1A).

**Figure 1.**
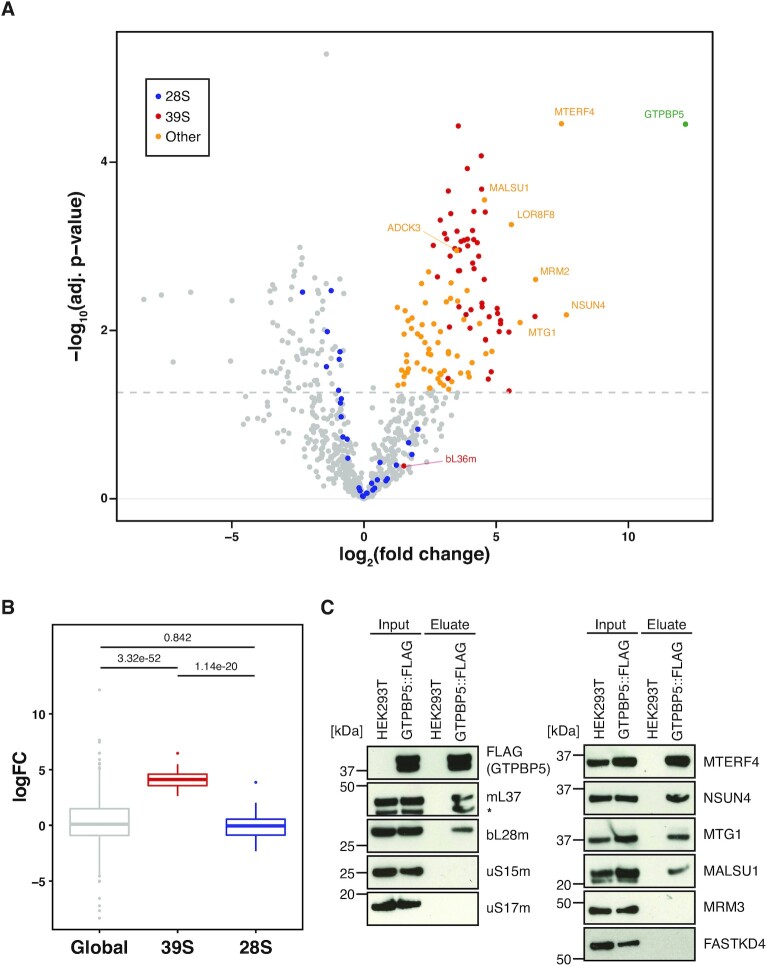
GTPBP5 interacts with the mt-LSU proteins and the MTERF4:NSUN4 in the mitoribosome assembly complex. (**A**) Quantitative mass spectrometry analysis of proteins interacting with GTPBP5::FLAG. Following FLAG-IP, eluates from HEK293T expressing GTPBP5::FLAG and control HEK293T without FLAG protein expression (WT) were subjected to label-free quantitative mass spectrometry (LFQ) (*n* = 3). Proteins with a positive log_2_(fold change) (logFC > 3) and –log_10_(*P*-value) of >1.3 (adjusted *P*-value < 0.05) are identified. Identified proteins are as indicated in the key. A fully annotated version of this dataset is presented as Supplementary Figure S2A and Table S5. (**B**) Boxplot comparison of the logFC of core proteins of the 39S (mt-LSU) or 28S (mt-SSU) relative to global proteins from dataset in A. Stated *P*-values indicate pair-wise significance of difference in logFC between global proteins, mt-LSU proteins or mt-SSU proteins as determined via Welch's unequal variances *t*-test. (**C**) Immunoblotting of GTPBP5::FLAG-IP. Input mitochondrial lysates and eluate of FLAG-IP from HEK293T expressing GTPBP5::FLAG and control HEK293T without FLAG protein expression (WT) were resolved via SDS-PAGE, western blotting was performed and subsequent membranes were probed with antibodies against FLAG and for proteins of either the mt-LSU (bL28m, mL37) or mt-SSU (uS15m, uS17m) (left panel), or for known mt-LSU assembly factors (right panel). The extra band in mL37 panel (*) represents NSUN4 signal from previous probing.

As part of our study, we aimed to assess the mitochondrial protein interactome of GTPBP5 to discern whether the previously observed *in vitro* interaction of GTPBP5 with the mt-LSU occurs in living cells. We therefore generated a HEK293T Flp-In T-REx cell line, which allows for expression of the GTPBP5::FLAG in a doxycycline inducible manner. The GTPBP5::FLAG overexpressing cells, together with the control HEK293T, without FLAG protein expression, were tested via confocal microscopy to confirm mitochondrial localization of GTPBP5 (Supplementary Figure S1B, upper and middle panel). Following 48 h of GTPBP5::FLAG expression, we combined mitochondrial isolation and FLAG-IP to assess GTPBP5 protein interactors through the application of label-free quantitative mass spectrometry, relative to a negative control (HEK293T without FLAG protein expression; *n* = 3) (Figure 1A). Through this approach, we observed enrichment of 51 out of 52 constituent proteins of the mt-LSU in the GTPBP5::FLAG pulldown (Figure 1A and Supplementary Figure S2A). Interestingly, the only protein of the mt-LSU not enriched was bL36m, known to be incorporated to the mt-LSU at a very late stage of assembly (7). Of particular note, we did not observe enrichment of mt-SSU core proteins over that of global proteins (Figure 1B), suggesting that GTPBP5 binds exclusively to the mt-LSU or mt-LSU late stage assembly intermediate and not to the 55S monosome particle.

Western blotting was performed for the input and eluate from the GTPBP5::FLAG IP experiments, which supported the specific interaction of GTPBP5::FLAG with constituent proteins of mt-LSU (mL37, bL28m), and not of the mt-SSU (uS15m, uS17m) (Figure 1C).

As a negative control, to exclude the possibility that mitoribosomal proteins may be non-specifically enriched during our FLAG-IP procedure, we performed FLAG-IP of mitochondrially-targeted luciferase (MtLuc::FLAG) under identical experimental conditions. We observed no enrichment of mt-LSU or mt-SSU proteins for our MtLuc::FLAG pulldown, assayed via western blotting (Supplementary Figure S2B), supporting the specific binding of GTPBP5 to the mt-LSU under these experimental conditions.

In addition to the enrichment of core mt-LSU proteins, GTPBP5::FLAG pulldown highly enriched known mt-LSU accessory factors, which have been implicated in mitoribosome biogenesis, including mitochondrial rRNA methyltransferase 2 (MRM2) (28,38), MTG1 (GTPBP7) (18), mitochondrial assembly of ribosomal large subunit 1 (MALSU1) (39,40) and L0R8F8 (7) (Figure 1A). MRM2 has been shown to perform 2′-O-ribose methylation at position U(1369) in 16S mt-rRNA (28,38), and its high enrichment here may suggest that GTPBP5 binds to the maturing mt-LSU at a similar stage of assembly to MRM2. In addition, MTG1 was recently found to bind to a late stage intermediate of mt-LSU assembly (18). Recently, Brown *et al.* have reported that MALSU1, together with L0R0F8 and mt-ACP, bind to the mt-LSU at the subunit interface to prevent premature formation of the 55S monosome particle (7). The high enrichment of these assembly factors (Figure 1A) further support the suggestion that GTPBP5 may bind to a late stage assembly intermediate of the mt-LSU.

Of particular note, we observed the greatest logFC in abundance of peptides related to NSUN4 (NOL1/NOP2/Sun domain family member 4) and MTERF4 (mitochondrial transcription termination factor 4). MTERF4 and NSUN4 have been found together to form a stoichiometric complex that binds to the mt-LSU and has been shown previously to be required for formation of the 55S monosome particle (41,25). Additional to its interaction with MTERF4, NSUN4 functions alone as a m^5^C rRNA methyltransferase, methylating the C841 residue in 12S mt-rRNA (40). In this role, NSUN4 would be expected to be bound to the mt-SSU, and therefore the enrichment observed for NSUN4 here is instead likely linked to its role in partnership with MTERF4, as we did not enrich for any mt-SSU proteins in our GTPBP5::FLAG-IP (Figure 1A, B). The high enrichment of both MTERF4 and NSUN4 upon GTPBP5::FLAG pulldown may indicate that GTPBP5 is binding to a mt-LSU particle that is nearing translational competency.

Western blotting was further used to confirm the specific enrichment of those mt-LSU assembly factors identified in LFQ proteomic analysis (MTERF4, NSUN4 MTG1, MALSU1, Figure 1C), whilst additional known mt-LSU interacting factors (e.g. MRM3, FASTKD4), were not identified via either LFQ or western blotting (Figure 1C). Through our immunoprecipitation analysis, therefore, we show that GTPBP5 interacts with the specific components of the late stage mt-LSU assembly intermediate within a biological system.

### GTPBP5 ablation results in a mitochondrial gene expression defect

**Figure 2.**
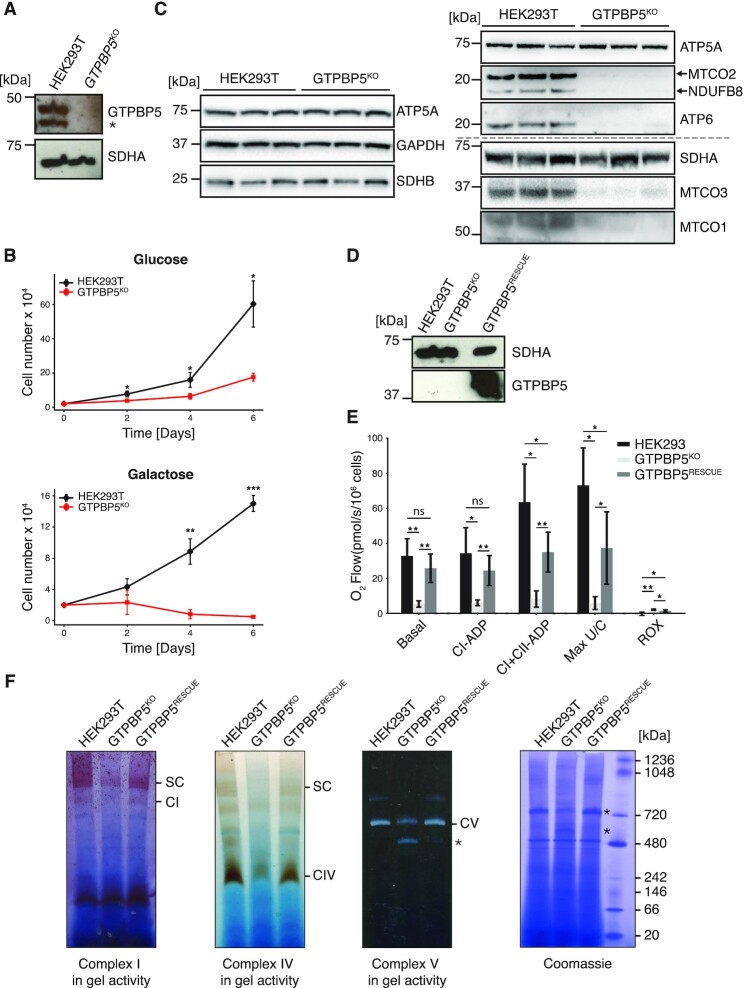
GTPBP5 knockout leads to alteration in mitochondrial gene expression. (**A**) Western blotting to confirm knockout of GTPBP5. Total lysate from HEK293T WT and GTPBP5^KO^ clones was resolved via SDS-PAGE, immunoblotting was performed and membranes were probed with anti-GTPBP5 antibody and anti-SDHA as a loading control. The asterisk represents a shorter version of GTPBP5 protein. (**B**) Proliferation of GTPBP5^KO^ cells is compromised. Growth curves of WT HEK293T, and GTPBP5^KO^ cells in DMEM containing either 4.5g/l glucose or 0.9 g/l galactose (three biological replicates were performed and mean average cell numbers at each time point is indicated, error bars = ±1 SD). Student's two-tailed *t*-test. *P*-values are as follows: (HEK293T versus GTPBP5^KO^) Glucose: 2d, 0.0227; 4d, 0.0241; 6d, 0.0241. Galactose: 2d, 0.405; 4d, 0.00944; 6d, 0.000997. (**C**) Western blotting to assess steady state levels of OxPhos subunits in GTPBP5^KO^. Total lysates from HEK293T and GTPBP5^KO^ cell lines were resolved via SDS-PAGE, immunoblotting was performed, and membranes were probed with OxPhos antibody cocktail against nuclear encoded proteins of complexes I (NDUFB8), complex II (SDHB) and complex V (ATP5A) and mtDNA-encoded complex IV protein MTCO2. In addition, membranes were probed with ATP6, MTCO3 and MTCO1 antibodies. GAPDH and SDHA were used as loading control. (C) Western blotting was performed on the control HEK293T (WT), GTPBP5^KO^ cells and KO cells expressing GTPBP5::FLAG (GTPBP5^RESCUE^) that were used for Oxygraph in (E). Antibody staining against FLAG was performed to confirm expression of the GTPBP5::FLAG protein. Complex II protein SDHA was used as loading control. (**D**) Mitochondrial respiration was measured in intact (basal) and in digitonin-permeabilized cells by the sequential addition of substrates and inhibitors to measure ADP-stimulated complex I respiration (CI-ADP; ADP, glutamate, malate, pyruvate) or ADP-stimulated complex I + II respiration (CI+II-ADP; + succinate), the complex I + II uncoupled maximal respiration rate (Max U/C; + CCCP) and the residual oxygen consumption (ROX; + rotenone, + antimycin A). Oxygen consumption is expressed in pmol of oxygen/s/10^6^ cells and measured by Oroboros oxygraph. Data is presented as mean ± 1SD (*n* ≥ 3). Significant differences between wild-type and KO or between wild-type and rescue cell lines were determined by two-tailed Student's *t* tests. *P < 0.05, **P < 0.01. Pairwise *P*-values are presented in the following format for each conditions (HEK293T WT versus GTPBP5^KO^, HEK293T WT vs GTPBP5^RESCUE^, GTPBP5^KO^ versus GTPBP5^RESCUE^), Basal: 0.00979, 0.297, 0.00367; CI-ADP: 0.0295, 0.2898, 0.00715; CI+CII-ADP: 0.0121, 0.0717, 0.00397; Max U/C: 0.0291, 0.0778, 0.0256; ROX; 0.00891, 0.0297, 0.0937). (**E**) BN-PAGE and in-gel activities of complex I, complex IV and complex V activities in mitochondrial protein extracts from HEK293T WT, GTPBP5^KO^ and GTPBP5^RESCUE^. Coomassie staining of the gel is shown to indicate equal loading. Asterisk in the complex V panel indicates accumulated F_1_-containing sub-complexes of complex V. Asterisks adjacent to the Coomassie staining panel highlight the amount of protein of complex V (upper asterisk) and of the F_1_-containing sub-complexes of complex V (lower asterisk). SC, Supercomplexes.

To further investigate GTPBP5 function, we produced a knockout cell line using plasmid-based CRISPR/Cas9 delivery. Two sgRNAs were designed to produce an out-of-frame deletion targeted to the first protein-coding exon of the *MTG2* gene, which encodes for the GTPBP5 protein (Supplementary Figure S3A). PCR analysis across the expected deletion site in our isolated GTPBP5 clone, revealed that the deletion was present on both alleles (Supplementary Figure S3B). To assess GTPBP5 expression in the produced knockout clone, we performed western blotting, which confirmed loss of expression in GTPBP5^KO^ (Figure 2A).

We assessed proliferation of GTPBP5^KO^ cells in high glucose medium, which resulted in a reduced rate of proliferation relative to that of control HEK293T cells (Figure 2B). We further cultured both lines in galactose medium, which forces cells to rely on OxPhos as their major source of ATP production. A striking inability of GTPBP5^KO^ cells to proliferate in galactose media was observed (Figure 2B), suggesting that a profound OxPhos defect is present in the GTPBP5^KO^ line.

We sought to further investigate the suspected OxPhos defect in the GTPBP5^KO^ line. Western blotting to assess levels of OxPhos complex proteins revealed a strong reduction in steady-state levels of Complex I protein NDUFB8 (nuclear-encoded), Complex IV proteins MTCO1, MTCO2 and MTCO3 (mtDNA-encoded) and Complex V protein ATP6 (mtDNA-encoded) whilst levels of nuclear-encoded SDHB (Complex II) and ATP5A (Complex V) were unaffected (Figure 2C).

To further support the specificity of the observed OxPhos phenotype to the loss of GTPBP5 expression, we derived a GTPBP5^KO^ line complemented with the *GTPBP5::FLAG* cDNA (GTPBP5^RESCUE^), which was stably introduced in a doxycycline-inducible manner using the Flp-In T-REx system. Expression of the GTPBP5::FLAG protein in the GTPBP5^RESCUE^ line was confirmed via western blotting (Figure 2D) and confocal microscopy (Supplementary Figure S1B, lower panel).

To understand the impact of GTPBP5 loss on mitochondrial bioenergetic function, we measured oxygen consumption. Mitochondrial respiration in the presence of Complex I or Complex II–linked substrates was significantly decreased in GTPBP5^KO^ cells (Figure 2E), consistent with the decreased steady-state protein levels of OxPhos subunit NDUFB8 (Figure 2C). Furthermore, mitochondrial oxygen consumption was partially recovered in GTPBP5^RESCUE^ cells (Figure 2E).

We next analysed in-gel activities of OxPhos complexes for control, GTPBP5^KO^ and GTPBP5^RESCUE^ lines (Figure 2F). BN-PAGE analysis revealed a reduction in activities of Complexes I, IV and V in their independent forms, and within supercomplexes in the case of Complexes I and IV (Figure 2F). In addition, for Complex V, we observed an accumulation of subassemblies with a reduced molecular mass relative to the F_1_F_0_ holoenzyme in the GTPBP5^KO^ line that retained ATP hydrolysis activity and represent F_1_-containing subcomplexes. Similar subassemblies are reported frequently where mtDNA expression is compromised (42–44).

Crucially, the reduction in activity of Complexes I, IV and V observed in the GTPBP5^KO^ line is rescued upon re-expression of GTPBP5::FLAG cDNA (Figure 2F), further supporting that the observed reduction in complex activities is specific to loss of GTPBP5, and likely is due to a general defect to mitochondrial gene expression.

### Ablation of GTPBP5 results in a severe mitochondrial translation defect

**Figure 3.**
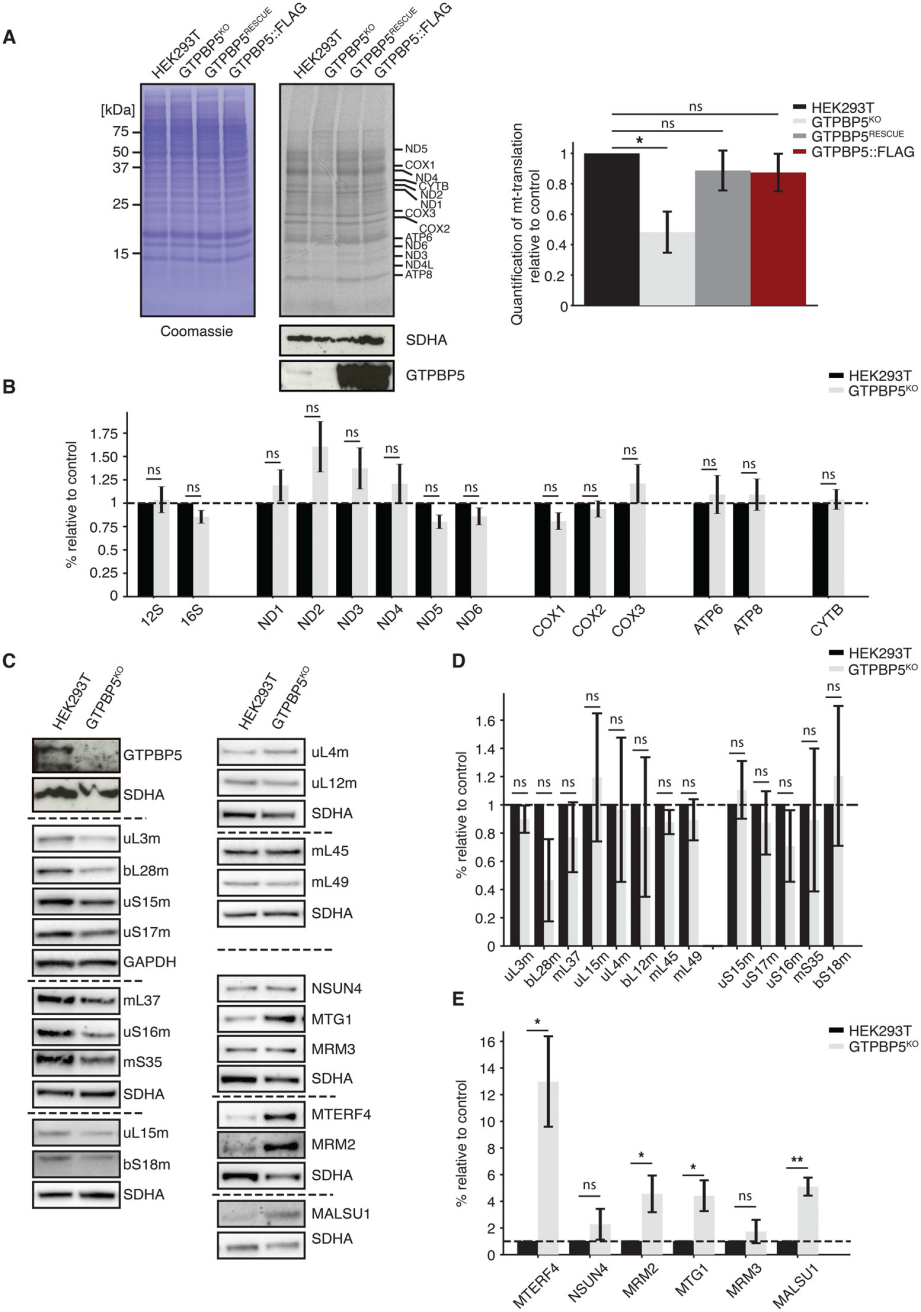
GTPBP5 knockout causes reduction in mitochondrial translation without affecting the steady-state levels of mitochondrial rRNAs/mRNAs and MRPs. (**A**) [^35^S]-labeling of mitochondrial translation in wild type HEK293T cells, GTPBP5^KO^ cells, GTPBP5^RESCUE^, and parental WT HEK293T cells overexpressing GTPBP5::FLAG. Following inhibition of cytosolic translation, cells were cultured for 30 minutes in the presence of a [^35^S]-methionine and cysteine mix to specifically label products of mitochondrial translation. Total cell extracts (30 μg) were resolved via 12% SDS-PAGE and visualized via autoradiography. Presented experiment is representative of three independent biological replicates. Relative quantification of [^35^S]-labelling signal (right panel) for the lane relative to Coomassie blue stain as loading control, using Image J. Mitochondrial translation in GTPBP5^KO^ cells is compared to HEK293T WT, GTPBP5^RESCUE^ and HEK293T expressing GTPBP5::FLAG, as in the left panel, for three independent biological replicates. Student's two-tailed t-test, *P*-value: 0.0219. (**B**) Quantitative real-time PCR to determine steady-state levels of mt-RNAs. Relative steady-state level comparison of mitochondrial transcripts in HEK293T WT cells and GTPBP5^KO^ cells by qPCR (*n* = 4 biological replicates, error bars represent SEM). Student's two-tailed *t*-test, *P*-values are as follows: (HEK293T vs GTPBP5^KO^), 12S: 0.807, 16S: 0.126, ND1: 0.335, ND2: 0.111, ND3: 0.189, ND4: 0.397, ND5: 0.0721, ND6: 0.0230, COX1: 0.124, COX2: 0.526, COX3: 0.379, ATP6: 0.681, ATP8: 0.626, CYTB: 0.730. (**C**) western blotting to assess steady-state levels of proteins of the mitoribosome and accessory factors. Total lysate from HEK293T WT and GTPBP5^KO^ clones was resolved via SDS-PAGE, immunoblotting was performed and membranes were probed with antibodies to the mt-LSU (uL3m, bL28m, mL37, uL15m, uL4m, uL12m, mL45 and mL49), mt-SSU (uS15m, uS16m, uS17m, mS35 and bS18m) and to accessory proteins to the mitoribosome (MTERF4, NSUN4, MTG1, MRM2, MRM3, MALSU1). Antibodies to SDHA and GAPDH were used to assess loading. (**D**) Quantification of steady-state levels of mitoribosomal proteins was performed from three biological replicates using ImageJ software, with normalization of signal of protein to either SDHA (mL37, uS16m, mS35, uL15m, bS18m, uL4m, uL12m, mL45, mL49) or GAPDH (uL3m, uS15m, bL28, uS17m). Student's two-tailed t-test, *P-* values are as follows: (HEK293T vs GTPBP5^KO^), uL3m: 0.2115, bL28m: 0.0863, mL37: 0.249, uL15m: 0.535, uL4m: 0.917, uL12m: 0.637, mL45: 0.131, mL49: 0.334, uS15m: 0.464, uS17m: 0.427, uS16m: 0.184, mS35: 0.749, bS18m: 0.548. (**E**) Quantification of steady-state levels of mitoribosome accessory factors was performed from three biological replicates using ImageJ software, with normalization of signal of protein to either SDHA (NSUN4, MALSU1, MRM3, MTG1) or GAPDH (MTERF4, MRM2). Student's two-tailed *t*-test, *P*-values are as follows: (HEK293T versus GTPBP5^KO^), MTERF4: 0.0256, NSUN4: 0.202, MRM2: 0.0471, MTG1: 0.036, MRM3: 0.297, MALSU1: 0.009.

We decided to further study mitochondrial translation within the GTPBP5^KO^ line and observed a substantial decrease in [^35^S]-Met/Cys labelled proteins derived from mitochondrial translation, to ∼50% of that observed in the HEK293T control line (Figure 3A). This defect in mitochondrial translation was rescued upon expression of GTPBP5::FLAG in the GTPBP5^RESCUE^ line, confirming that the translation defect is linked to loss of GTPBP5 expression. Of note, induction of GTPBP5 re-expression was performed with 50 ng/ml doxycycline (as described in 37,39), leading to very high GTPBP5 expression levels (Figures 2D and 3A), however, this did not alter significantly mitochondrial translation. The same observation was made when testing mitochondrial translation in GTPBP5::FLAG overexpressing cell line (Figure 3A). Nevertheless, we cannot exclude that high overexpression of GTPBP5 might, to some extent, inhibit protein synthesis, as previously observed for ObgE (23).

Next, we investigated the steady-state levels of mt-mRNAs by quantitative real-time PCR (Figure 3B) and northern blotting (Supplementary Figure S4A). No major changes in stability of the transcripts were detected, apart from the increased steady-state levels of ND2, suggesting that the general translation defect was not caused by a lowered availability of mitochondrial RNAs to translate.

We therefore sought to explore whether the mitochondrial translation defect observed in the GTPBP5^KO^ line was linked to a defect in the mitoribosome. Steady-state analysis of mt-LSU and mt-SSU proteins revealed no significant reduction in the levels of mt-LSU and mt-SSU proteins overall, although some (bL28m, mL37) were mildly affected (Figure 3C, D).

Steady-state level analysis of mt-LSU accessory factors, including those enriched in our GTPBP5::FLAG pulldown experiments, revealed an upregulation in steady-state levels of a number of factors (Figure 3C, E), with the most striking increase observed for MTERF4, which was highly enriched in our GTPBP5::FLAG-IP experiment (Figure 1A). The increase in steady-state levels of several factors may reflect a compensatory mechanism to promote mt-LSU assembly under conditions of absence of GTPBP5. The lack of significant increase in NSUN4 levels, however, is notable, as it is thought to function as a heterodimer with MTERF4 on the mitoribosome to promote monosome formation.

### Loss of GTPBP5 results in a defect in monosome formation

**Figure 4.**
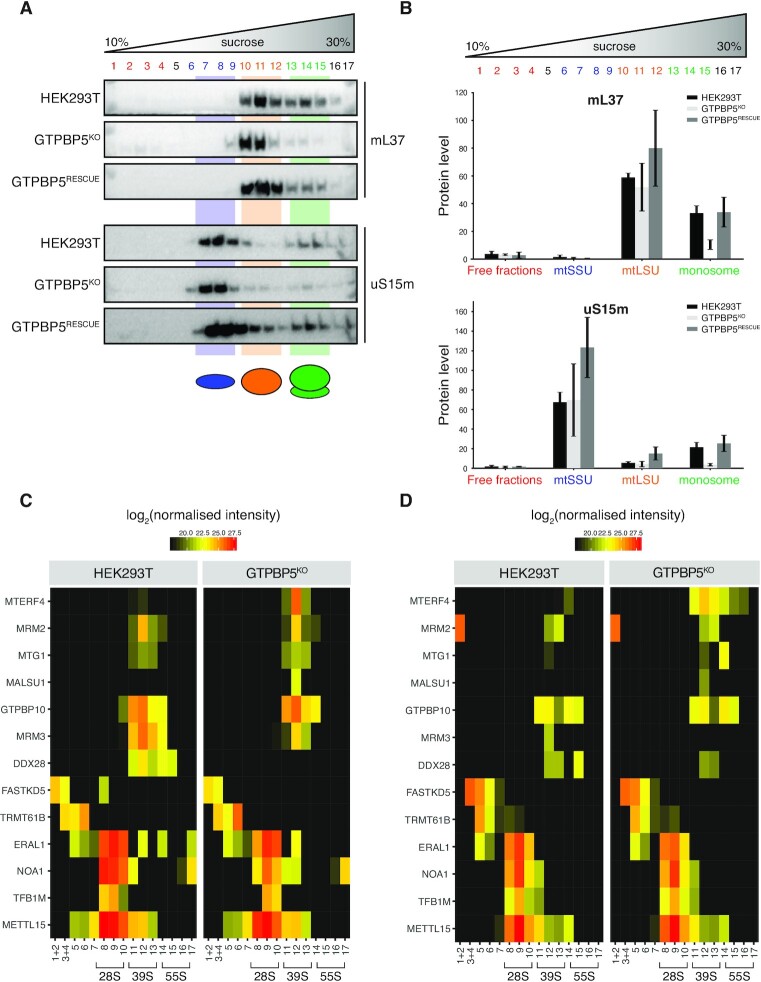
GTPBP5 is important for monosome formation and its absence leads to accumulation of some late-stage mt-LSU assembly factors. (**A**) Sedimentation of mitochondrial ribosomes on 10–30% isokinetic sucrose gradients for HEK293T control, GTPBP5^KO^ and GTPBP5^RESCUE^. Mitochondria were isolated from cells, and lysates were loaded onto gradients. Following centrifugation, obtained fractions were analysed by western blotting with antibodies against proteins of the mt-LSU (mL37), the mt-SSU (uS15m). (**B**) Quantification of the gradient distribution of mitoribosomal components (mL37 and uS15m) in HEK293T WT, GTPBP5^KO^ and GTPBP5^RESCUE^ cell lines. Measurements were performed using ImageJ software, indicating averages for three independent biological experiments. The *y*-axis represents the fraction of the total signal in each set of fractions relative to the total signal for HEK293T WT gradient. Error bars represent SEM. (**C**) SILAC-based proteomic analysis of mitoribosome assembly factors in sucrose gradient fractions. Heatmap of the identified assembly factors associated with the mt-LSU and mt-SSU were plotted as log2 transformed and normalized intensity values as indicated colours. (**D**) Heatmap plotted as described in (C) for the label swap experiment.

To further investigate the role of GTPBP5 in mitoribosome function, we performed sucrose gradient density fractionation of mitochondrial lysates derived from HEK293T control, GTPBP5^KO^ and GTPBP5^RESCUE^ lines followed by western blot analysis. Through this, we observed a reduction in the quantity of 55S monosome particles in GTPBP5^KO^, whilst expression of the GTPBP5::FLAG protein recovered the abundance of 55S particles (Figure 4A, B and Supplementary Figure S5A). In addition, we performed sucrose gradient centrifugation analysis followed by northern blotting to investigate 16S and 12S mt-rRNAs migration and observed a decrease of both rRNA levels in the monosome fractions in GTPBP5^KO^ cells, consistent with the Western Blot results (Supplementary Figure S5B). This suggests that GTPBP5 is important for mt-LSU assembly, with loss of GTPBP5 stalling the developing mt-LSU in a state which reduces its capacity to bind to the mt-SSU.

To better determine the stage of mt-LSU assembly at which GTPBP5 acts and therefore the potential mt-LSU intermediate that accumulates in its absence, we combined sucrose gradient centrifugation experiment with stable isotope labelling with amino acids in cell culture (SILAC). This allowed us to measure quantitative changes in the steady-state levels of assembly factors associated with the mt-SSU and mt-LSU. GTPBP5^KO^ cells in combination with HEK293T cells or GTPBP5^RESCUE^ cells were grown in media with ‘light’ amino acids or stable ‘heavy’ isotope labelled amino acids. Cells were pooled and isolated mitochondria were subjected to sucrose gradient centrifugation with collected fractions analysed by mass spectrometry. The mitoribosome assembly factors identified were quantified using the observed intensities of the relative peptides. Comparison of HEK293T sample with GTPBP5^KO^ revealed an increase in MTERF4 levels associated with the mt-LSU in GTPBP5^KO^, as well as a slight increase of other mt-LSU-associated assembly factors such as MTG1 and MALSU1 (Figure 4C, D). MRM2 and GTPBP10 levels associated with the mt-LSU did not change between control and GTPBP5^KO^. Interestingly, DDX28 and MRM3 levels were decreased in GTPBP5^KO^ samples compared to control, suggesting they might be involved in different steps of assembly, not trapped in GTPBP5^KO^ (Figure 4C,D). The levels of FASTKD5 and TRMT61B as well as some of the mt-SSU assembly factors (ERAL1, NOA1, TFB1M, METTL15) remained largely unchanged (Figure 4C, D). Next, we extended our analysis by comparing GTPBP5^RESCUE^ sample with GTPBP5^KO^. We did not observe major changes in mt-LSU-associated levels of MTERF4 between the two samples, nor several other factors (e.g. NSUN4 (previously not detected), MTG1, GTPBP10) (Supplementary Figure S6A, B), suggesting that the factors trapped in GTPBP5^KO^ may also interact with the mt-LSU assembly intermediate containing GTPBP5.

### Interaction of GTPBP5 with the mt-LSU requires GTP hydrolysis

Previously, we observed an enrichment of core proteins of the mt-LSU upon GTPBP10::FLAG-IP when lysates were pre-incubated with non-hydrolysable GTP analog (GMPPNP) (Supplementary Figure S7B, and previously reported in Busch *et al.* (19)). This indicated that GTP hydrolysis is required for release of GTPBP10 from the maturing mt-LSU, in line with what has been observed for the homologous ObgE protein in *B. subtilis* (44).

**Figure 5.**
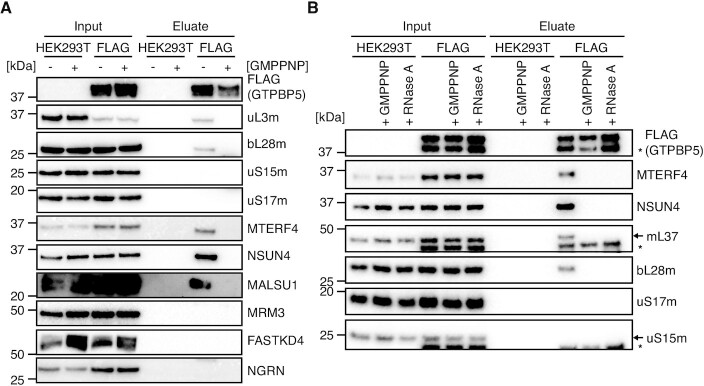
GTP hydrolysis activity of GTPBP5 is required for binding to mitochondrial ribosomes and MTERF4:NSUN4 module. (**A**) FLAG-IP of GTPBP5 in the presence and absence of non-hydrolysable GTP analog, GMPPNP. FLAG-IP was performed from HEK293T expressing GTPBP5::FLAG and control HEK293T, where mitochondrial lysates were treated with or without GMPPNP (20 mM) during the lysis stage prior to IP protocol. Resulting input, mitochondrial lysates, and eluates were resolved via SDS-PAGE, western blotting was performed and subsequent membranes were probed with antibodies against FLAG and for proteins of either the mt-LSU (uL3m, bL28m) or mt-SSU (uS15m, uS17m). In addition, immunoblotting was performed for known accessory factors of the mt-LSU (MTERF4, NSUN4, MALSU1, MRM3, FASTKD4 and NGRN). (**B**) FLAG-IP as in (A), where mitochondrial lysates were treated with or without RNase A (20 μg/ml), or with GMPPNP (20 mM), during lysis prior to IP protocol. Immunoblotting was performed with antibodies against FLAG and for proteins of either the mt-LSU (mL37, bL28m) or mt-SSU (uS15m, uS17m), and mt-LSU accessory factors MTERF4 and NSUN4. The extra band in GTPBP5 panel (indicated with *) might represent a shorter version of GTPBP5 protein, with its MTS removed, while the one in uS15m panel (*) a possible degradation product of GTPBP5. The extra band in mL37 panel (*) represents the FLAG signal from previous probing.

To determine if a similar mechanism of action is present for GTPBP5, we performed GTPBP5::FLAG pulldown experiment in the presence and absence of GMPPNP. In stark contrast to GTPBP10::FLAG, pre-incubation with GMPPNP prior to GTPBP5::FLAG-IP resulted in a complete loss of interaction of the mt-LSU subunits with GTPBP5::FLAG, as assessed via western blotting (Figure 5A, B and Supplementary Figure S7A), suggesting that GTP hydrolysis is important for GTPBP5 binding to the mt-LSU, not for its release. Similar results were observed when we preincubated the lysates with GTP instead of GMPPNP, with a loss of interaction with the mt-LSU/MTERF4:NSUN4 for GTPBP5 and an enhanced interaction with the mt-LSU for GTPBP10 (Supplementary Figure S7A, B). Notably, GTPBP10 was not found to bind to MTERF4 in any of the conditions tested (Supplementary Figure S7B), reinforcing the hypothesis of a specific link between GTPBP5 and MTERF4:NSUN4 complex.

To further confirm the importance of the G-domain for GTPBP5 function, we designed a S238A mutant form of GTPBP5::FLAG protein at a highly conserved position in the Walker A motif in the G-domain (Supplementary Figure S7C) (45). GTPBP5^S238A^::FLAG pull-down experiment showed no interaction of GTPBP5^S238A^ with the mt-LSU, nor with MTERF4 (Supplementary Figure S7D).This is in line with the GMPPNP experiments suggesting that the interaction is dependent on GTP hydrolysis.

To further assess the importance of GTP hydrolysis for GTPBP5 function, we generated a GTPBP5^KO^ line complemented with the *GTPBP5^S238A^::FLAG* cDNA (GTPBP5^RESCUE(S238A)^), which was stably introduced in a doxycycline-inducible manner using the Flp-In T-REx system, as previously performed for the generation of GTPBP5^RESCUE^ cell line. GTPBP5^RESCUE(S238A)^ cells were grown in galactose media and displayed a growth rate comparable to GTPBP5^KO^ cells, which was significantly lower when compared to HEK293T and GTPBP5^RESCUE^ cells (Supplementary Figure S7E). Additionally, a decrease in mitochondrial protein synthesis was observed in GTPBP5^RESCUE(S238A)^ and GTPBP5^KO^ cells compared to HEK293T and GTPBP5^RESCUE^ (Supplementary Figure S7F), therefore confirming that this mutation is important for GTP hydrolysis and consequently for GTPBP5 function.

To investigate the nature of GTPBP5 interaction with MTERF4 and NSUN4, we performed FLAG-IP from mitochondrial lysate, which had been pre-treated with RNase A prior to IP. Upon RNase A treatment, we anticipated that the rRNA within the mitoribosome would be degraded and that mt-LSU proteins would dissociate (42). Under these conditions, we observed a lack of enrichment of mt-LSU proteins upon GTPBP5::FLAG pulldown (mL37, bL28m) (Figure 5B), which are clearly enriched in the absence of RNase A treatment (Figures 1 and 5A). In addition, the enrichment of accessory factors MTERF4 and NSUN4 was also lost (Figure 5B), suggesting that the interaction of GTPBP5 with the MTERF4:NSUN4 complex relies on the integrity of the mt-LSU or that of a late-stage mt-LSU intermediate.

## DISCUSSION

Despite recent breakthroughs in our structural understanding of the mitoribosome and the identification of a number of accessory factors which have been implicated in mitoribosome biogenesis, the complete repertoire of required factors is likely far from complete and the mechanistic details of the function of already known factors need to be revealed. Here, we confirm that Obg-superfold family protein GTPBP5 can be added to the growing list of factors whose function relates to the maturation of the mt-LSU.

During the review of our manuscript, a characterization of GTPBP5 has been reported by Maiti *et al.* (25). Consistent with our data, Maiti et al. also found that GTPBP5 is involved in the late stages of the mtLSU assembly and GTPBP5 knock out affects mitoribosome maturation and consequently mitochondrial translation.

### The role of Obg family GTPases in ribosome assembly

The presence of two Obg family GTPases in human mitochondria raises questions related to whether each protein has a divergent role in mt-LSU assembly and whether they bind to exclusive positions on the mt-LSU. Alignment of their primary sequences to that of *E. coli* ObgE, indicates an overall high level of conservation (Supplementary Figure S8A), as also shown in previous studies (20,21). However, whilst loops 1, 2 and 3 of the Obg domain in GTPBP5 are predicted to structurally resemble those of ObgE (Supplementary Figure S8B), closer inspection of the primary sequence of GTPBP5 in loops 1 and 3 reveals differences in the key basic residues which have previously been shown in ObgE to be required for LSU binding, likely through direct rRNA coordination to the peptidyl transferase center (PTC) in 50S LSU (23) (shown in bold and underlined in Supplementary Figure S8A). This may reflect a distinct binding mechanism of GTPBP5 to the LSU compared to ObgE. Moreover, comparisons between the predicted GTPBP10, GTPBP5 and the resolved ObgE structures reveal distinct deletions and expansions, in the Obg- and G-domains, respectively, of GTPBP10 (Supplementary Figure S8A,B). Also, the C-terminal domains (CTD) of each protein's primary sequence are highly divergent (Supplementary Figure S8A, not shown in the structural predictions due to their intrinsically disordered nature) (23,36). Together, these differences may reflect on functional divergence between GTPBP5 and GTPBP10.

The role of Obg family member ObgE (*E. coli*) and other bacterial homologues in ribosome assembly has been extensively characterized and may provide clues to the action of GTPBP5 on the maturing mt-LSU (23,36,37,47,48). ObgE has been implicated in binding to a late stage assembly of the bacterial LSU (49), causing conformational changes at the interface of the 50S that are likely to prevent association of the LSU with the SSU, precluding 70S formation (23).

Similar to ObgE, GTPBP5 appears to be excluded from the 55S monosome, evidenced by a lack of pulldown of components of the mt-SSU in our GTPBP5-IP (Figure 1). Moreover, bL36m, a protein incorporated at the very last stages of mt-LSU assembly (7) is also missing from the GTPBP5 pull-down experiments. Therefore, GTPBP5 is likely to be involved in the very late stages of assembly, and must be released before 55S monosome particles are formed.

Both GTPBP5 or GTPBP10 were found to functionally complement a loss of ObgE protein in *E. coli* (50). However, through our investigation of GTPBP5 function, and through previous studies by ourselves and others (19–21), we observed that the loss of function of either GTPBP5 or GTPBP10 results in a severe defect in mitochondrial translation, suggesting that whilst they are both GTPases of the Obg-superfamily, GTPBP5 and 10 have functions which are not redundant, although both are likely related to late stage maturation of the mt-LSU. Notably, with our experimental set up, we observed a 50% reduction of translation in GTPBP5^KO^ cells, while the steady state levels and activities of the OxPhos components were more drastically reduced. A similar phenotype has been observed in the case of GTPBP10 deficiency (20) and in the recent study of GTPBP5 by Maiti et al., where it was shown that while COX2 and COX3 steady-state levels were not detectable by western blot analyses, mitochondrial translation rates were decreased only by 50–60% (25). Taking that into consideration, we cannot rule out additional role of GTPases, downstream of mitoribosome maturation.

Mitoribosome biogenesis is not necessarily a linear process, and although both GTPBP5 and GTPBP10 are thought to bind to late state intermediates of the mt-LSU, it is unclear whether they function in sequential steps in maturation, or in concert at different regions of the complex. It is intriguing to note that no published datasets of pulldown of GTPBP10, or our presented data of GTPBP5, revealed reciprocal pulldown of the two Obg GTPases, despite both proteins being implicated in binding to late stage assemblies of the mt-LSU. It cannot be excluded, therefore, that the Obg domain of either protein may facilitate binding to the same site on the maturing mt-LSU, in a mutually exclusive manner, at different stages of assembly.

We are currently limited to structural predictions of GTPBP5 and GTPBP10. However, recent advances in cryo-EM structures have allowed the structural interrogation of assembly factors in concert with the mitoribosome (7). Obtaining similar structures of either GTPBP5 or GTPBP10 bound to the mt-LSU particle would allow direct determination of the binding sites of either protein in the maturing mt-LSU, including insight into how the interaction of the Obg-domain is coordinated with the mt-LSU and whether binding induces conformational changes to the mt-LSU, in a similar manner to ObgE.

### Mechanism of GTP hydrolysis of GTPBP5

**Figure 6.**
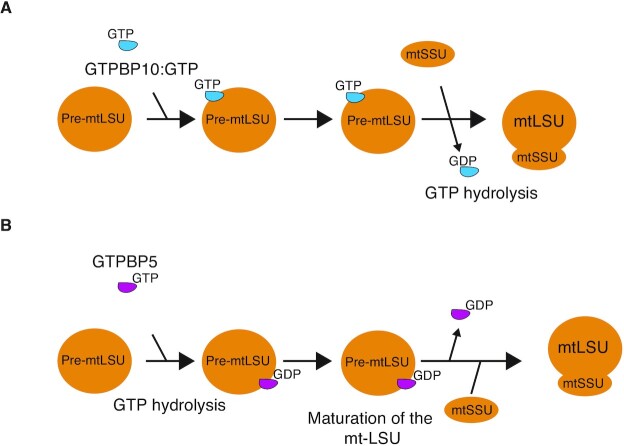
Model for GTPases action during mt-LSU biogenesis. (**A**) Mechanism of action of GTPBP10 during mitoribosome biogenesis. GTP-bound GTPBP10 binds to the premature mt-LSU and GTP hydrolysis is required for release of GTPBP10 from the mt-LSU. During this step, the energy released during GTP hydrolysis can potentially be used for driving conformational changes of the mitoribosome and/or binding/release of other assembly factors. (**B**) Mechanism of action of GTPBP5. GTP-bound GTPBP5 binds to a late-stage intermediate of the mt-LSU in a manner that requires GTP hydrolysis. The energy released during GTP hydrolysis can potentially be used for driving conformational changes of the mitoribosome. During later stages, GTPBP5 dissociates from the mt-LSU.

There are conformational differences between the GTP and GDP-bound forms of Obg-family GTPases, due to alteration in the switch elements of the G-domain (51), which allows for alterations in the angle between the Obg fold and the G-domain itself. This conformational change between different guanine nucleotide-bound states may be of particular significance for the role of both GTPBP5 and GTPBP10 in aiding conformational changes to mt-LSU. Here in, we found that pre-incubation of mitochondrial lysates with non-hydrolysable GMPPNP led to a loss of interaction of GTPBP5 with the mt-LSU under our experimental conditions (Figure 5A, B and Supplementary Figure S7A). This was a surprising finding, since it has previously been shown that for the homologous ObgE, GTPase activity is stimulated when it is already bound to the LSU (23), with GTP hydrolysis linked to the release of ObgE from the LSU particle. Also, GTPBP10 shows an increased interaction with the mt-LSU following pre-incubation with GMPPNP prior to FLAG-IP (19) (Supplementary Figure S7B), indicating that GTP hydrolysis is linked to GTPBP10 release from the mt-LSU (Figure 6A). Interaction of another GTPase, NOA1 (C4orf14), with the mt-SSU has also been described to be dependent on GTP in a similar manner to ObgE and GTPBP10 (14). The behavior of GTPBP5 seems instead to reflect a necessity for GTP hydrolysis in promoting GTPBP5 binding to the ribosome itself (Figure 6B), suggesting that GTPBP5 association with the mt-LSU might induce potential conformational changes important for mitoribosome maturation. GTPBP5 release from the mt-LSU may instead be mediated through the action of additional protein factors, or through additional conformational changes in the mt-LSU at a later stage in assembly.

### Interplay of GTPBP5 with other mt-LSU assembly factors

The enrichment of both MALSU1:L0R8F8 and MTERF4:NSUN4 complexes with the mt-LSU particle obtained via GTPBP5::FLAG pulldown strongly supports the notion that GTPBP5 is interacting with a late stage mt-LSU assembly that is reaching maturity.

The increased steady-state levels of MTERF4 and its accumulation in the mt-LSU-containing sucrose gradient fractions observed in the GTPBP5^KO^ line is a potential compensatory effect to overcome the loss of function of GTPBP5 (Figures 3C, E and 4C, D). It is intriguing that whilst MTERF4 and NSUN4 are required to function together to promote monosome formation (40), steady-state levels of NSUN4 is not upregulated in GTPBP5^KO^, perhaps indicating that NSUN4 is not a limiting factor in the formation of the MTERF4:NSUN4 complex, and therefore upregulation of NSUN4 steady-state levels is not likely to provide a compensatory effect. This compensatory upregulation of MTERF4 and other accessory factors may explain why there is residual monosome formation and translation in the absence of GTPBP5. On the contrary, DDX28 levels decreased in GTPBP5^KO^ compared to control, suggesting that this factor is not associated with GTPBP5^KO^ mt-LSU intermediate, and may possibly act downstream in the assembly pathway (Figure 4C, D). Of note, when comparing GTPBP5^KO^ with GTPBP5^RESCUE^, no major changes in the mt-LSU-associated assembly factors levels were observed, consistent with our co-IP experiments showing association of GTPBP5 with MTERF4:NSUN4 complex, MRM2, MALSU1 and MTG1.

The mechanisms of overexpression of accessory factors alleviating defects in ribosome assembly have previously been observed, indicating that ribosome assembly is not a linear process. For example, ObgE overexpression in *E. coli* was able to rescue ribosome assembly in a null mutation of RrmE/RrmJ/FtsJ (ribosomal RNA large subunit methyltransferase E), likely through stabilization of the conformation of maturing 50S subunits, without the requirement to rescue the methylation status of 23S rRNA (52). Similarly, through experiments performed in yeast, overexpression of Mtg2p, the yeast homologue of GTPBP5, was found to recover the deleterious effect on mitochondrial translation of a model lacking the rRNA methyltransferase Mrm2p (24), the yeast homologue of RrmE.

In addition to MTERF4:NSUN4, in our GTPBP5::FLAG pulldown experiments we detected high enrichment of the mitochondrial rRNA methyltransferase MRM2 (Figure 1). Furthermore, we observed increased levels of MRM2 in GTPBP5^KO^ cells and a clear association with the GTPBP5^KO^ mt-LSU intermediate (Figures 3C, E and 4C, D). Whilst we do not focus on the possible interplay between the functions of the GTPBP5 protein and MRM2 methyltransferase on the 16S mt-rRNA in this study, it is notable, as mentioned above, that in yeast the homologue of GTPBP5 was able to partially rescue *MRM2* knockout (24). It is possible, therefore, that GTPBP5 functions in a similar way in the human mt-LSU, to promote adoption of a conformation that allows MRM2 binding or methylation of 16S. Cryo-EM analysis previously revealed that ObgE binds to a site adjacent to the methylation site of FtsJ (23S rRNA position U2552, PDB: 4CSU) (23), the bacterial homolog of MRM2. Importantly, in recently published study by Maiti *et al.*, BioID experiments confirmed a strong interaction of MRM2 and GTPBP5 (25). Moreover, the levels of methylation of the 16S rRNA at positions U1369 and G1145 that are normally catalysed by MRM2 and MRM3, respectively, were shown to be partially reduced in the absence of GTPBP5 (25), suggesting an important interplay between MRM2 and GTPBP5.

Together, interaction of GTPBP5 with several assembly factor may play a role in quality control mechanisms, promoting ordered maturation of the mt-LSU during last stages of mitoribosome biogenesis.

## DATA AVAILABILITY

Proteomic data from SILAC-based sucrose gradient analysis are deposited in ProteomeXchange, PXD017384.

## Supplementary Material

gkaa1131_Supplemental_File
