# Supplementary material for: Human GTPBP5 is involved in the late stage of mitoribosome large subunit assembly
Source: Nucleic Acids Res. 2020 Dec 7;49(1):354–70. doi: 10.1093/nar/gkaa1131 (PMC7797037; doi:10.1093/nar/gkaa1131)
Supplement: gkaa1131_Supplemental_File [file gkaa1131_supplemental_file.pdf]

Supplementary information for

Human GTPBP5 is involved in the late stage of mitoribosome large subunit assembly

Miriam Cipullo, Sarah F. Pearce, M. Isabel G. Lopez Sanchez, Shreekara Gopalakrishna, Annika Krüger, Florian Schober, Jakob D. Busch, Xinping Li, Anna Wredenberg, Ilian Atanassov, Joanna Rorbach

SUPPLEMENTARY FIGURES

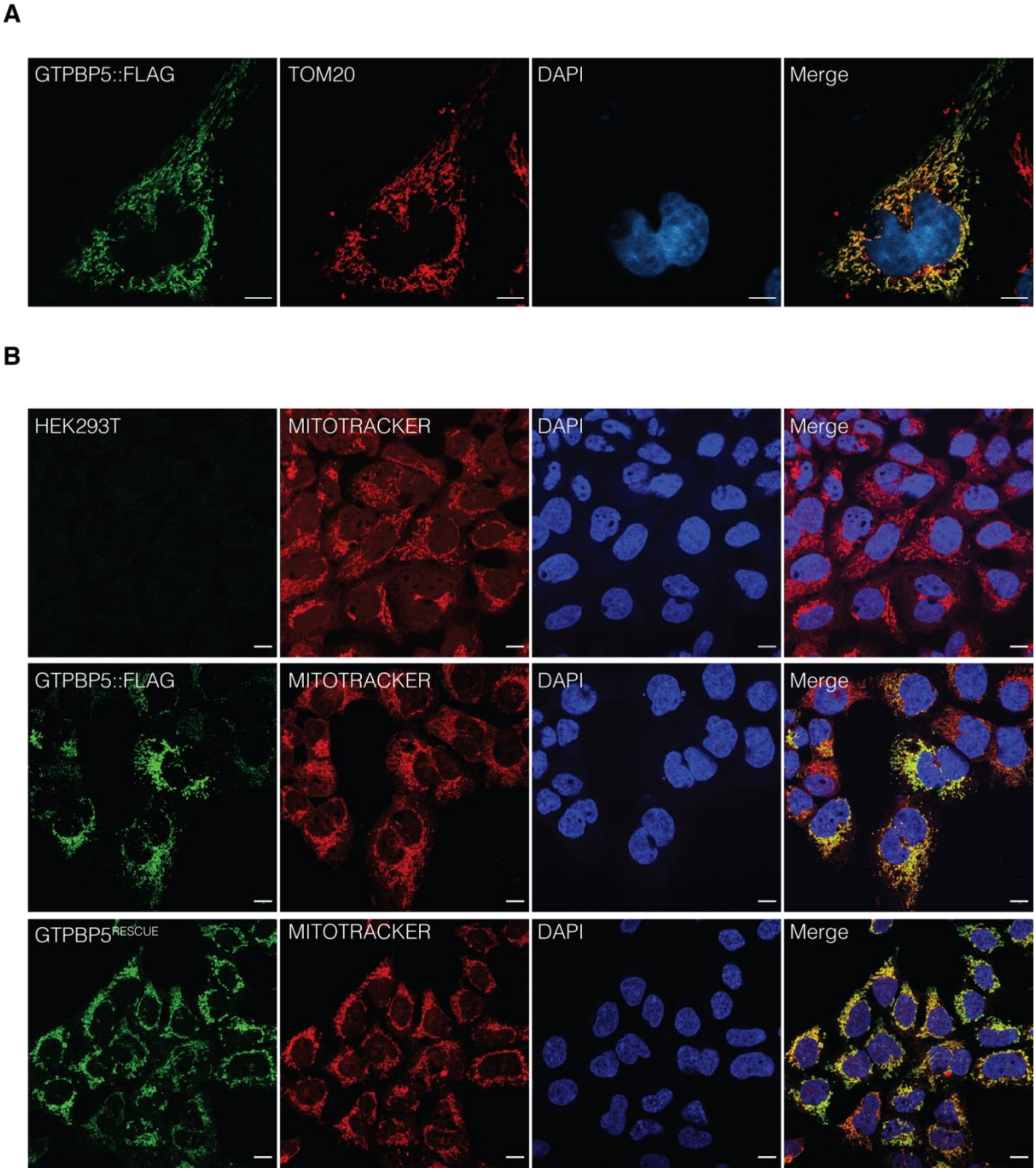

Supplementary Figure S1. Intracellular localization of GTPBP5 via immunocytochemistry. (A) *GTPBP5::FLAG* cDNA was transiently transfected into HOS cells. Cell nuclei were stained with DAPI (blue). The GTPBP5::FLAG protein product was detected via an anti-FLAG antibody and visualized using anti-mouse secondary antibody conjugated to Alexa Fluor 488 (green). Mitochondria were visualized using anti-TOM2O

antibody, an integral protein of the outer mitochondrial membrane, and anti-rabbit secondary antibody conjugated to AlexaFluor 568. A digitally merged image of DAPI, GTPBP5::FLAG and TOM20 signals reveals colocalization of GTPBP5::FLAG with the mitochondrial network. **(B)** GTPBP5::FLAG was detected in HEK293T control line (upper panel), WT HEK293T cells overexpressing GTPBP5::FLAG (middle panel) and GTPBP5^{RESCUE} cells (lower panel) under 50 ng/ml doxycycline induction. The GTPBP5::FLAG signal was detected via an anti-FLAG antibody, whereas mitochondria were visualized using MitoTracker Red. Secondary antibodies were used as in **(A)**.

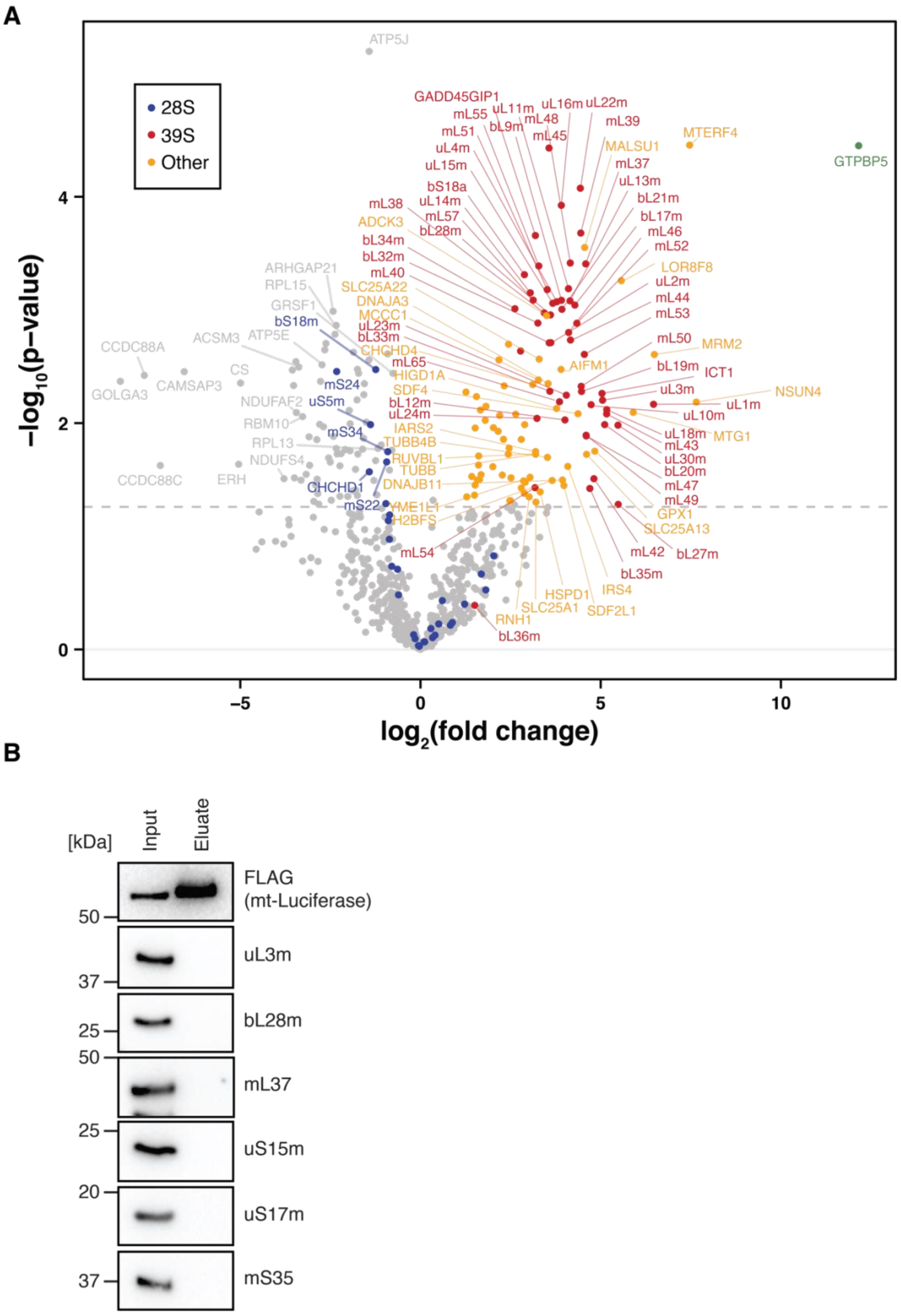

Supplementary Figure S2. **GTPBP5 interactome. (A)** Quantitative mass spectrometry analysis of GTPBP5::FLAG interactome following FLAG-immunoprecipitation and using

HEK293T as control. Proteins with a log_2(fold change) (logFC) of greater than 3 and $-log_{10}$(p-value) of greater than 1.3 are labelled. Several negative hits are labelled, including all MRPs belonging to the mt-SSU. bL36m that is not enriched by GTPBP5-IP is also highlighted. **(B)** FLAG-immunoprecipitation of mitochondrially-targeted luciferase as a negative control for binding of mitoribosomal proteins. Resulting lysates and eluates were resolved via SDS-PAGE, immunoblotting was performed and membranes were probed with antibodies to proteins of the mt-LSU (uL3m, bL28m, mL37) and mt-SSU (uS15m, uS17m, mS35).

A

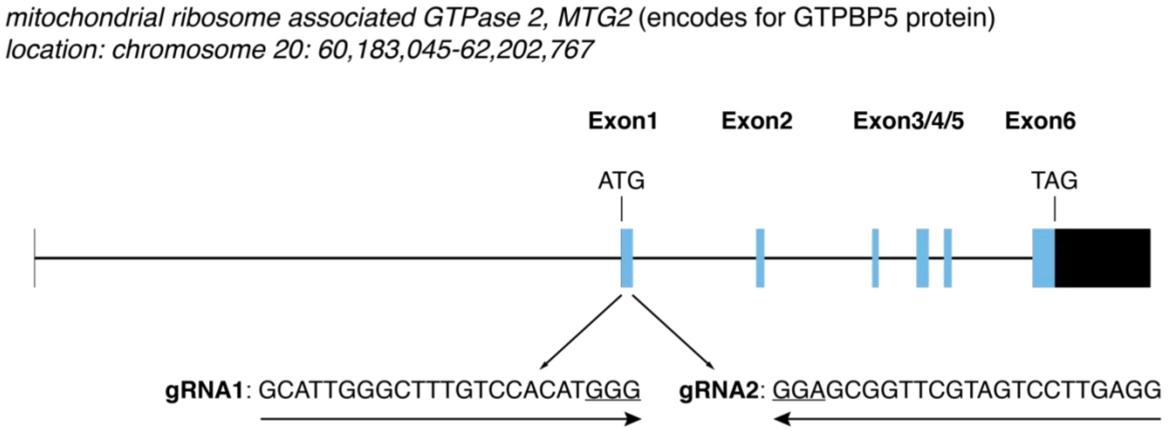

B

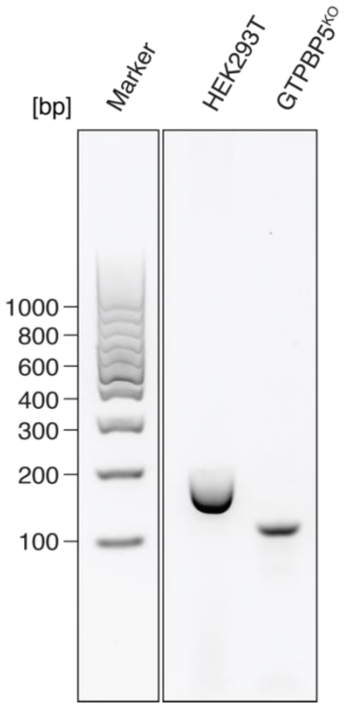

Supplementary Figure S3. Generation of GTPBP5 knockout clone. (A) Schematic representation of MTG2 gene locus indicating target sites of gRNAs without protein-coding exon 1. Underlined sequences represent the PAM sites. **(B)** PCR analysis performed targeting *MTG2* exon 1 gRNAs cutting sites.

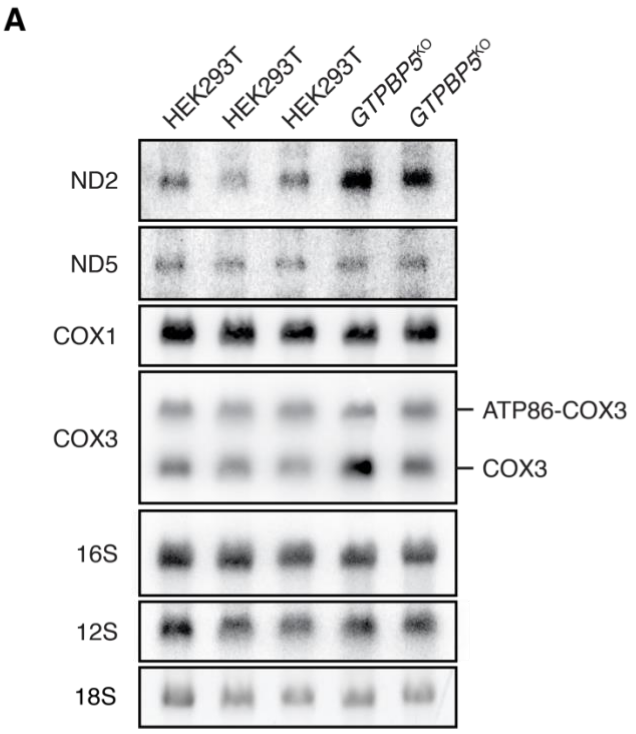

Supplementary Figure S4. mt-rRNAs and mt-mRNAs steady state levels assessed by Northern Blot analysis. (A) Northern blotting to assess steady state levels of mt-rRNAs and mt-mRNAs in GTPBP5^{KO} line. Nuclear encoded 18S rRNA is used as loading control.

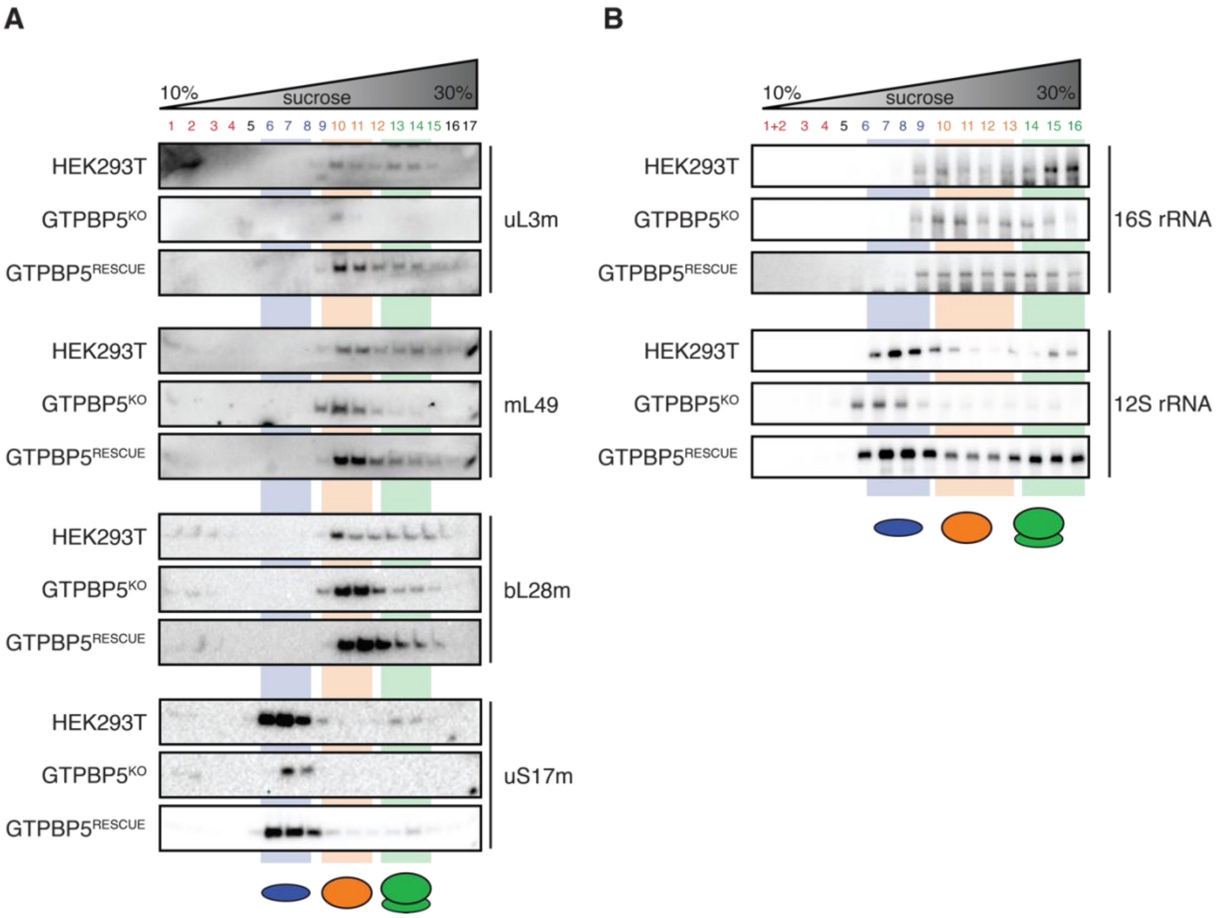

Supplementary Figure S5. GTPBP5 co-sediments with the mt-LSU in GTPBP5^{RESCUE} cell line and is important for monosome formation. (A) Sucrose gradient centrifugation analysis performed on HEK293T control, GTPBP5^{KO} and GTPBP5^{RESCUE} cell lines. Mitolysates were loaded onto 10-30% isokinetic sucrose gradients and following centrifugation, obtained fractions were analyzed by western blotting with antibodies against uL3, mL49, bL28m, uS17m. **(B)** Sucrose gradient centrifugation analysis performed on HEK293T control, GTPBP5^{KO} and GTPBP5^{RESCUE} followed by Northern Blot analysis. Mitolysates were loaded as in **(A)** and, following centrifugation, 16 fractions were analyzed by Northern Blotting with probes against 16S and 12S mt-rRNA. Fraction 1 and 2 were merged prior to the Northen Blot analysis.

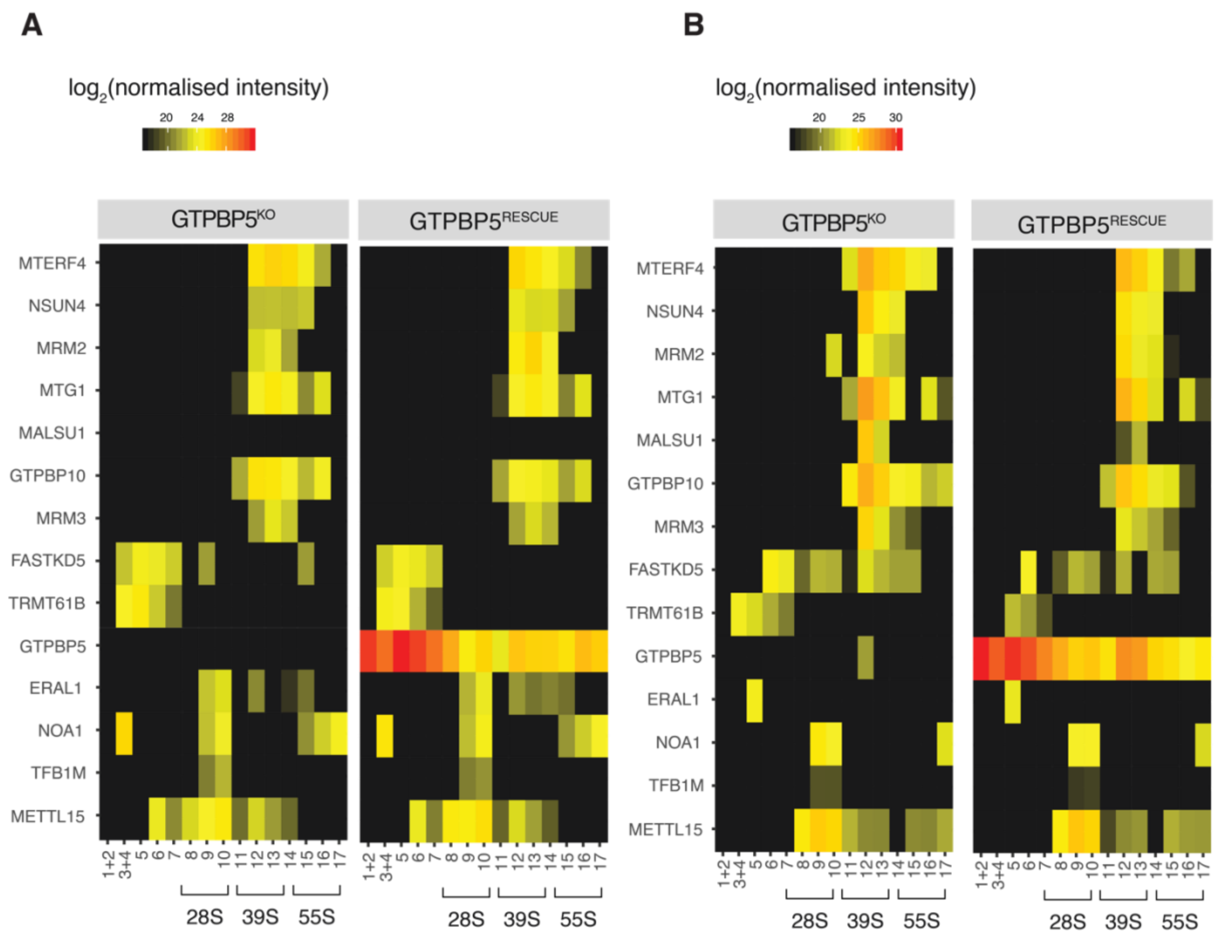

Supplementary Figure S6. Analysis of mt-LSU intermediate in GTPBP5^{KO} cells in comparison with GTPBP5^{RESCUE} cells. (A) SILAC-based proteomic analysis of mitoribosome assembly factors in sucrose gradient fractions. Heatmap of the identified assembly factors associated with the mt-LSU and mt-SSU were plotted as log2 transformed and normalised intensity values as indicated colours. **(B)** Heatmap plotted as described in **(A)** for the label swap experiment. Of note, GTPBP5 signal in GTPBP5^{KO} most likely comes from non-labelled overexpressed GTPBP5 in GTPBP5^{RESCUE}.

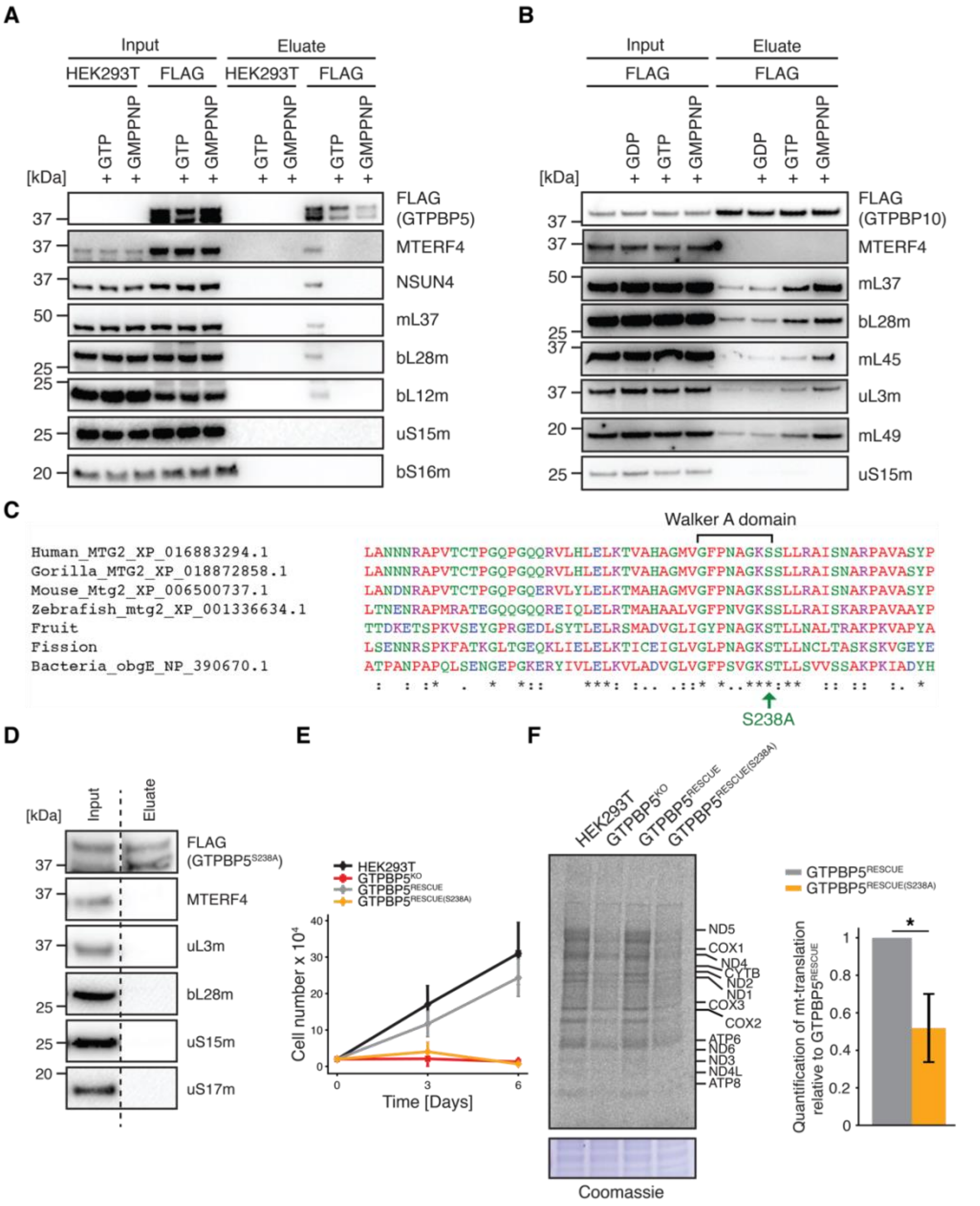

Supplementary Figure S7. GTPBP5 and GTPBP10 interactions with the mt-LSU in the presence of GTP and GMPPNP. (A) Immunoblotting of GTPBP5::FLAG IP in the presence and absence of GTP and GMPPNP. Input mitochondrial lysates, pre-incubated with or without GTP (20 mM) and GMPPNP (20 mM), and eluates of FLAG-IP from HEK293T expressing GTPBP5::FLAG and control HEK293T without FLAG protein expression (WT) were resolved via SDS-PAGE, western blotting was performed and subsequent membranes were probed with antibodies against FLAG and proteins of either the mt-LSU (mL37, bL28m, bL12m) or mt-SSU (uS15m, bS16m). In addition, membranes were probed for MTERF4 and NSUN4. **(B)** Immunoblotting of GTPBP10::FLAG IP in the presence and absence of GDP, GTP and

GMPPNP. Input mitochondrial lysates were pre-incubated with or without GDP (20 mM), GTP (20 mM) and GMPPNP (20 mM). Input and eluates were analysed as in A. **(C)** Generation of GTPBP5 mutant. The mutation was designed to be inserted in the highly conserved S238 residue of the Walker A domain as described in a previous study (46). **(D)** Immunoblotting of $GTPBP5^{S283A}$::FLAG IP. Input mitochondrial lysates and eluate of FLAG-IP from HEK293T expressing $GTPBP5^{S283A}$::FLAG were analysed as described in **(A)** and **(B)**. **(E)** Growth curves measurements of WT HEK293T, $GTPBP5^{KO}$, $GTPBP5^{RESCUE}$ and $GTPBP5^{RESCUE(S238A)}$ cells in DMEM containing 0.9 g/l galactose (three biological replicates were performed and mean average cell numbers at each time point is indicated, error bars = +/- 1 SD). Student's two-tailed t-test. *P*-values are as follows: (HEK293T vs $GTPBP5^{KO}$): 3d, 0.028; 6d, 0.021. ($GTPBP5^{RESCUE}$ vs $GTPBP5^{KO}$): 3d, 0.049; 6d, 0.010. ($GTPBP5^{RESCUE(S238A)}$ vs $GTPBP5^{KO}$): 3d, 0.223; 6d, 0.501. (HEK293T vs $GTPBP5^{RESCUE(S238A)}$): 3d, 0.013; 6d, 0.027. (HEK293T vs $GTPBP5^{RESCUE}$): 3d, 0.067; 6d, 0.081. **(F)** [^{35}S]-labelling of mitochondrial translation in wild type HEK293T, $GTPBP5^{KO}$, $GTPBP5^{RESCUE}$ and $GTPBP5^{RESCUE(S238A)}$ cells. Following inhibition of cytosolic translation, cells were cultured for 30 minutes in the presence of a [^{35}S]-methionine and cysteine mix to specifically label products of mitochondrial translation. Total cell extracts (30 μg) were resolved via 12% SDS-PAGE and visualised via autoradiography. Presented experiment is representative of three independent biological replicates.

Relative quantification of $GTPBP5^{RESCUE}$ and $GTPBP5^{RESCUE(S238A)}$ samples [^{35}S]-labelling signal (Right panel) for the lane relative to Coomassie blue stain as loading control, using Image J. Mitochondrial translation in $GTPBP5^{RESCUE(S238A)}$ cells is compared to $GTPBP5^{RESCUE}$, as in the right panel, for three independent biological replicates. Student's two-tailed t-test, *P*-value: 0.0443.

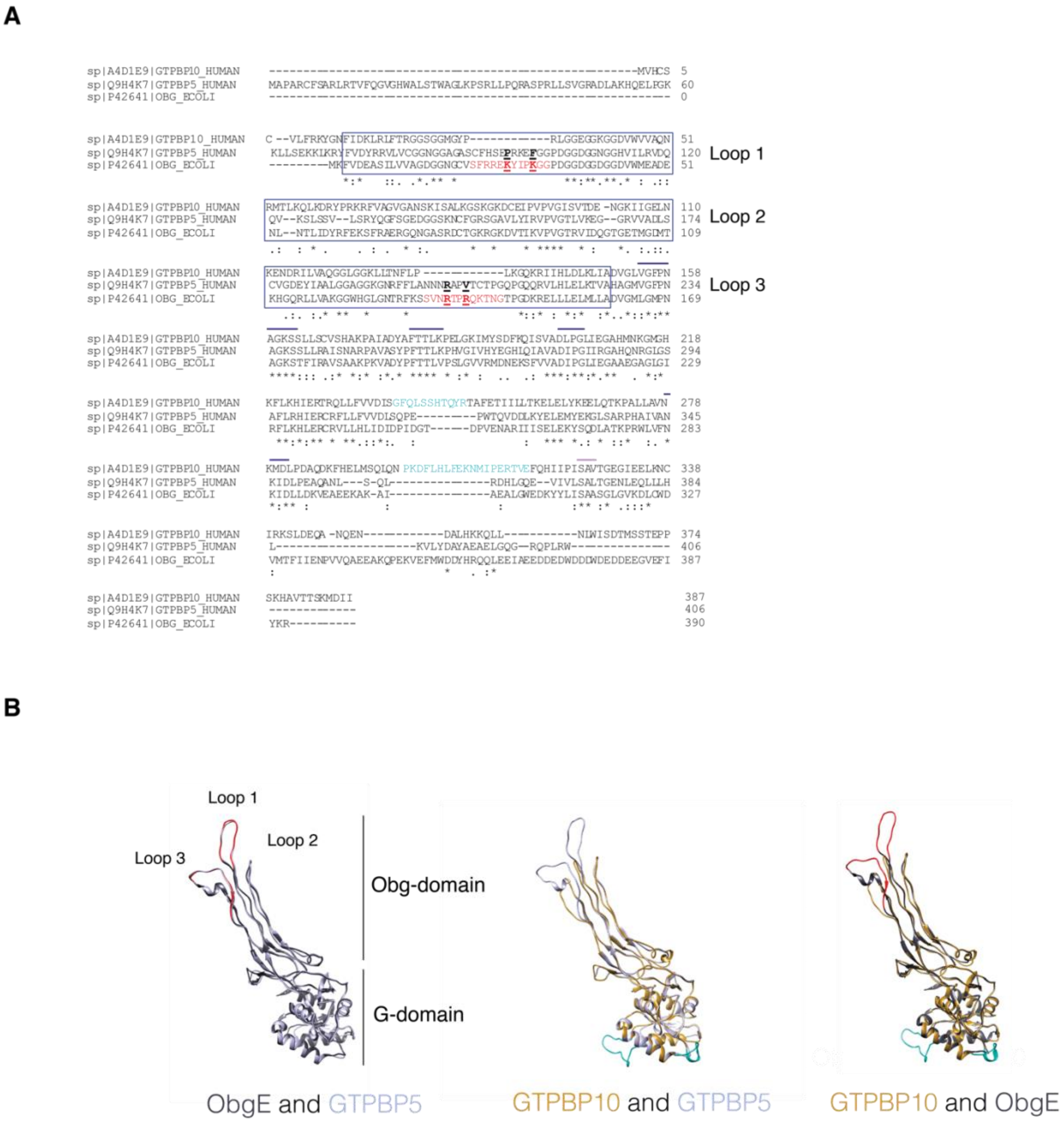

Supplementary Figure S8. GTPBP5 belongs to the Obg-subfamily. (A) Primary sequence alignment of *E. coli* ObgE and human GTPBP5 and GTPBP10 using Clustal OMEGA. Uniprot identifiers used: GTPBP10: A4D1E9, GTPBP5: Q9H4K7, ObgE: P42641. Major structural domains in the proteins are indicated. Key residues within ObgE, which are required for rRNA coordination are indicated in bold and are underlined. Residues contributing to structural elements, which are present in ObgE loops 1 and 3 but absent from GTPBP10 are colored in red, and residues contributing to structural elements, which are present in GTPBP10 G-domain but absent from ObgE are colored in cyan. **(B)** Predicted structural comparisons of human Obg family proteins GTPBP5 and GTPBP10 with *E. coli* ObgE. Structural predictions of both

human Obg family proteins were generated using SWISS-MODEL using the *E. coli* ObgE protein as a template (PDB: 4CSU) (52). Molecular graphics and analyses were performed with UCSF Chimera, developed by the Resource for Biocomputing, Visualization, and Informatics at the University of California, San Francisco, with support from NIH P41-GM103311 (53). Red and cyan coloring of specific protein portions as in **(A)**.

SUPPLEMENTARY TABLES

Supplementary Table 1. List of primers used for gene cloning

**Primer Sequence (5’→3’)**	
BamHI_GTPBP5_Fwd	CTTTCTTGGATCCATGGCACCTGCAAGGTGTTTC
GTPBP5_FLAG_XhoI_Rev	CTTTCTTTCTCGAGCTACTTATCGTCGTCATCCTT GTAATCCCACCTGAGCGGCTGGCG
GTPBP5_S238A_Fwd	ACGCCGGGAAGGCCTCACTGCTCC
GTPBP5_S238A_Rev	GGAGCAGTGAGGCCTTCCCGGCGTT

Supplementary Table 2. List of Antibodies

**Antibody**	**Company**	**Catalog No.**
Anti-FLAG	Abcam	ab1257
GTPBP5	Proteintech Group	20133-1
GAPDH	Abcam	Ab8245
SDHA	Abcam	ab14715
MRPL3/uL3m	Prestige Antibodies, Sigma-Aldrich	HPA043665
MRPL37/mL37	Prestige Antibodies, Sigma-Aldrich	HPA025826
MRPL12/bL12m	Prestige Antibodies, Sigma-Aldrich	HPA022853
MRPL28/bL28m	Prestige Antibodies, Sigma-Aldrich	HPA030594
MRPL15/uL15m	Proteintech Group	18339-1
MRPL4/uL4m	Prestige Antibodies, Sigma-Aldrich	HPA051261
MRPL49/mL49	Proteintech Group	15542-1
MRPL45/mL45	Proteintech Group	15682-1
MRPS16/uS16m	Prestige Antibodies, Sigma-Aldrich	HPA054538

MRPS35/mS35	Proteintech Group	16457-1
MRPS15/uS15m	Proteintech Group	17006-1
MRPS17/uS17m	Proteintech Group	18881-1
MRPS22/mS22	Proteintech Group	10984-1
MRPS18B/bS18m	Proteintech Group	16139-1
OXPHOS human WB	Abcam	ab110411
MTCO1	Invitrogen	459600
MTCO3	Abcam	Ab110259
ATP6	Proteintech Group	55313-1
TOM20	Santa-Cruz	sc-11415
MTERF4	Prestige Antibodies, Sigma-Aldrich	HPA027097
NSUN4	invitrogen	720212
MTG1	Prestige Antibodies, Sigma-Aldrich	HPA037827
MRM3/RNMTL1	Prestige Antibodies, Sigma-Aldrich	HPA023292
MALSU1	Prestige Antibodies, Sigma-Aldrich	HPA020487
FASTKD4/TBRG4	abcam	ab99317
MRM2/FTSJ2	Abcam	ab60068
NGRN	Proteintech Group	14885-1
HRP secondary rabbit	GE Healthcare	NA9340V
HRP secondary mouse	GE Healthcare	NA9310V
HRP secondary goat	Santa-Cruz	sc-2354

Supplementary Table 3. List of primers for rRNA and mRNA probe preparation

**Primer Sequence (5’→3’)**	
12S Fwd	CACTGAAAATGTTTAGACGGG
12S Rev	GGCTCCTCTAGAGGGATATG

16S Fwd	TAGATATAGTACCGCAAGGG
16S Rev	GACTTGTTGGTTGATTGTAG
ND2 Fwd	TCCCAGAGGTTACCCAAG
ND2 Rev	GAGTAGTGTGATTGAGGTGGAG
ND5 Fwd	GTAGCATTGTTCGTTACATGG
ND5 Rev	ACTGCTGCGAACAGAGTG
CO1 Fwd	CTTATTCGAGCCGAGCTG
CO1 Rev	GGTATAGAATGGGGTCTCCTC
CO3 Fwd	CCACCAATCACATGCCTATC
CO3 Rev	ACGTGAAGTCCGTGGAAGCC

Supplementary Table 4. List of TaqMan probes (Invitrogen)

TaqMan probe Assay ID	
ACTB	Hs99999903
MT-ND1	Hs02596873
MT-ND2	Hs02596874
MT-ND3	Hs02596875
MT-ND4	Hs02596876
MT-ND5	Hs02596878
MT-ND6	Hs02596879
MT-CYTB	Hs02596867
MT-COX1	Hs02596864
MT-COX2	Hs02596865

MT-COX3	Hs02596866
MT-ATP6	Hs02596862
MT-ATP8	Hs02596863
MT-RNR1	Hs02596859
MT-RNR2	Hs02596860
